# Chiral Hypervalent, Pentacoordinated Phosphoranes

**DOI:** 10.3390/molecules21111573

**Published:** 2016-11-21

**Authors:** Dorota Krasowska, Jacek Chrzanowski, Piotr Kiełbasiński, Józef Drabowicz

**Affiliations:** 1 Centre of Molecular and Macromolecular Studies, Polish Academy of Sciences, Sienkiewicza 112, 90-363 Lodz, Poland; piokiel@cbmm.lodz.pl; 2 Department of Chemistry, Environment Protection and Biotechnology, Jan Dlugosz University in Czestochowa, Armii Krajowej Ave. 13/15, 42-200 Czestochowa, Poland

**Keywords:** hypervalency, chirality, phosphoranes, Berry pseudorotation, turnstile rotation

## Abstract

This review presents synthetic procedures applied to the preparation of chiral (mainly optically active) pentacoordinated, hypervalent mono and bicyclic phosphoranes. The mechanisms of their stereoisomerization and their selected interconversions are also presented.

## 1. Introduction

The phenomenon of chirality plays a very important role in organic chemistry and biological processes [1,2,3,4,5]. Therefore an easy access to chiral compounds, especially optically active species, constitutes a vital challenge in modern synthetic organic chemistry. At least two reasons are responsible for this fact. Firstly, single stereoisomers are used very often as active components in formulations which serve as drugs, food additives or substances having the requested flavor or fragrance. The second reason stems from their application as chiral catalysts in various asymmetric reactions [6,7]. Moreover, detailed mechanistic description of a number of reactions would not be possible without taking into account their stereochemical aspects. These can be studied only with the use of optically active species which allow simultaneous application of kinetic and polarimetric measurements carried out with the use of the suitably designated model substrates (enantiomerically or diastereomerically pure or enriched samples) containing an appropriate chirality element [8]. In the organophosphorus chemistry the phenomenon of chirality is very common among organic derivatives having different valency and/or coordination number [9,10]. Their optical activity can be related to the presence of the following elements of chirality:

(a) a stereogenic center (for trivalent, tricoordinated, tetravalent tetracoordinated, pentavalent tetracoordinated derivatives)
(b) “trigonal bipyramidal chirality”
(c) “tetragonal bipyramidal chirality”

**Figure 1:**
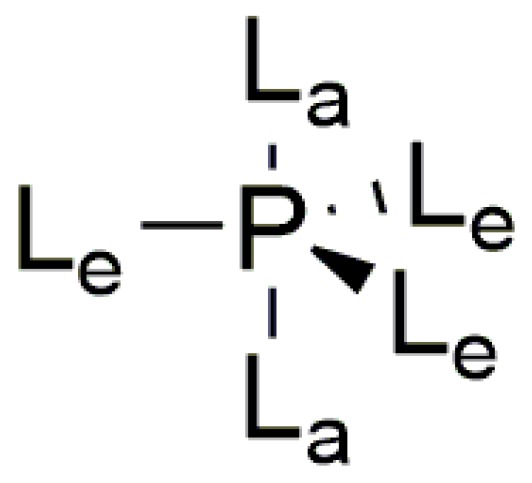
General formula of a phosphorus-containing trigonal bipyramid.

Organophosphorus derivatives in which optical activity results from the presence of a phosphorus atom at a center of trigonal bipyramid, are commonly named phosphoranes [11,12]. They have the general formula P(L_a_)_2_(L_e_)_3_, where “a” means an axial position and “e” means an equatorial position (Figure 1).

**Figure 2:**
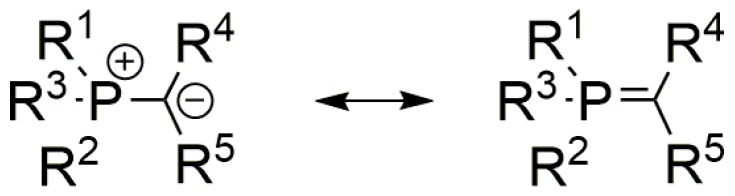
Resonance structure of phosphonium ylides.

They constitute the family of hypervalent derivatives, showing “trigonal bipyramidal chirality” in which the central phosphorus atom has expanded its valence shell from 8 to 10 electrons (the concept was for the first time proposed by Musher in 1969) [13]. According to general systematic scheme proposed by Martin and coworkers [14,15] [N-P-L (_n_A_m_B) coding system, in which N stands for the number of valence electrons associated formally with a central phosphorus atom and L shows the number of ligands (A and B stand for the bonding element)]—this group should be considered as the family of 10-P-5 derivatives. The definition of hypervalency is also formally fulfilled by the corresponding phosphonium ylides (in which a phosphorus atom has also expanded its formal valence shell from 8 to 10 electrons). However, when one considers that the important resonance structures of these compounds are polarized from phosphorus to carbon it becomes evident that they represent 8-P-4 species (Figure 2). Therefore they do not meet the condition of hypervalency and they are excluded from this review.

The nature of bonding of hypervalent derivatives is presented in details in a few chapters of “*The Chemistry of Hypervalent Compounds*” [16,17] and more recently has again been studied using the topological analysis of the electron localization function (ELF)” [18]. Their structure and reactivity are dominated by the weak three-center-four-electron (3c-4e) bond. Its weakness results from the fact that two of the four electrons that participate in the bonding are in a nonbonding molecular orbital.

Phosphoranes, like other compounds having a trigonal bipyramide (TBP) geometry can exist in an enantiomeric form when the number of different ligands is sufficiently large to induce chirality of such a structure. For 10-P-5 derivatives having the general structure with five substituents (Figure 1) bonded to the central phosphorus atom the numbers of achiral and chiral structures are given in Table 1. According to the Mutterties rule [19] in hypervalent structures the more electronegative ligands tend to occupy the apical positions whereas the lone electron pair “should be located at the equatorial position” [19]. Moreover, “five- and six-membered rings, which stabilize the hypervalent molecules, span both the axial and equatorial positions” [14].

**Figure 3:**
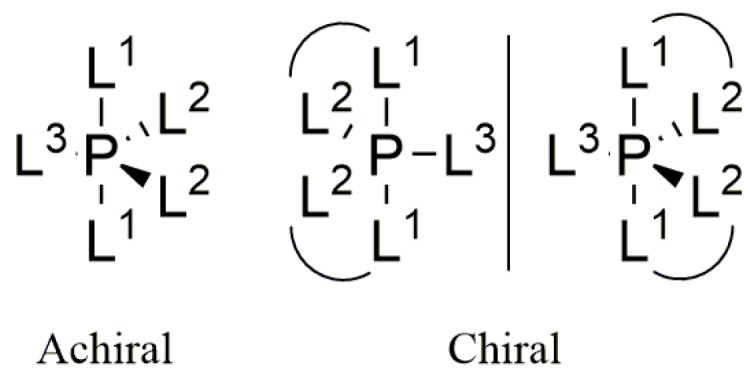
Chiral and achiral structure of hypervalent phosphoranes.

It is evident from this table that phosphoranes containing at least three different ligands can be chiral and for structures PL^1^_2_L^2^_2_L^3^ chirality may appear in the symmetric bicyclic spiro derivatives (Figure 3). Therefore, from the synthetic point of view, hypervalent structures exhibiting chirality due to the presence of one or two bidentate ligands should be more easily available than its acyclic analogues.

**Scheme 1:**
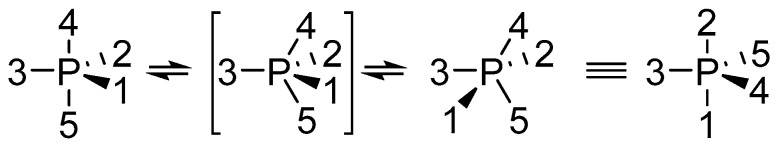
Berry pseudorotation mechanism.

**Scheme 2:**
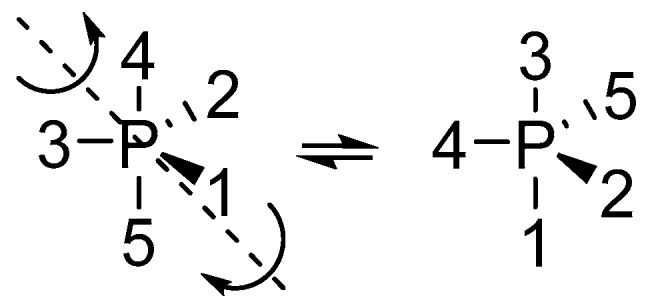
“Turnstile” rotation mechanism (TR).

In hypervalent phosphoranes 10-P-5 the apical and equatorial ligands can be interchanged with each other according to the Berry pseudorotation mechanism (BPR) [20,21,22] (Scheme 1) or by the “turnstile” rotation mechanism (TR) proposed by Ugi and Ramirez [23,24,25,26] (Scheme 2). It should be noted here that the very recent DFT calculations of the fluxional behavior of experimentally known pentavalent molecules have suggested the equivalence of the turnstile rotation with the Berry pseudorotation. This suggestion is based on identification of three principal mechanisms by which the substituents interchange can be achieved (Berry pseudorotation, threefold cyclic permutation, and half-twist axial-equatorial interchange) [27].

As early as 1949, Wittig and Rieber [28] reported isolation of pentaphenylphosphorane as the first example of a pentacoordinated organophosphorus derivative. However, a real opening in the hypervalent organophosphorus chemistry can be ascribed to a much later finding that the Ramirez reaction affords oxyphosphoranes by an oxidative addition of α-diketones to trivalent phosphines [29,30] and Westheimer’s proposal that a pentacoordinate phosphorane (able to stereomutate by pseudorotation) is involved in the hydrolysis of phosphoric esters [31]. Since that time, studies devoted to the synthesis, structural determinations, stereochemistry and reactivity of this group of the organophosphorus derivatives, have been carried out in a number of academic and industrial laboratories. Their results are regularly published in renowned chemical journals and, fortunately for those who work on these topics, are regularly presented in the chapters devoted to penta- and hexacoordinated organophosphorus derivatives included in the subsequent volumes of RSC monographic series “*Organophosphorus Chemistry*” [32,33,34,35,36,37,38,39,40,41,42,43]. In this respect, a very recent review highlighting methods for the asymmetric synthesis of P-chiral pentacoordinated spirophosphoranes should also be mentioned [44].

## 2. Chiral Hypervalent Phosphoranes and Their Anions

### 2.1. Chiral Phosphoranes as Reactive Intermediates

**Scheme 3:**
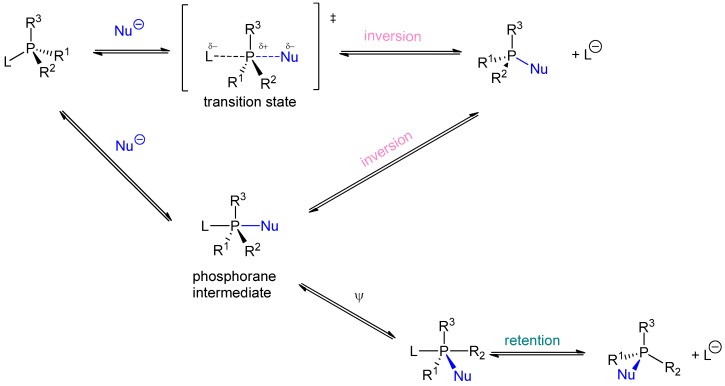
Possible mechanism for nucleophilic substitution reaction at phosphorus.

In most cases the papers describing the involvement of unstable chiral phosphoranes as reactive intermediates tackle the problem of the mechanism of S_N_-P reactions. This is connected with the fact that a nucleophilic substitution at phosphorus can occur either synchronously according to an S_N_2-P mechanism or in a stepwise manner by an addition-elimination mechanism (A-E). The later involves formation of a phosphorane as an intermediate, by the addition of a nucleophile_s_ (Nu) to a chiral substrate. It is now generally accepted that diaxial or diequatorial disposal of entering “Nu” and leaving “L” groups in a trigonal bipyramidal structure of a transient or intermediary phosphorane should lead to inversion of the configuration at phosphorus while the steric course of axial-equatorial substitution (which is possible if a phosphorane intermediate undergoes pseudorotation) is predicted to be a retention (Scheme 3).

The stereochemical consequences (retention or inversion) of such substitution reactions may be conveniently discussed with the aid of the Desargues–Levi graph [45], which originally was proposed by Mislow [46] for stereoisomerization of pentacoordinate phosphoranes.

**Scheme 4:**
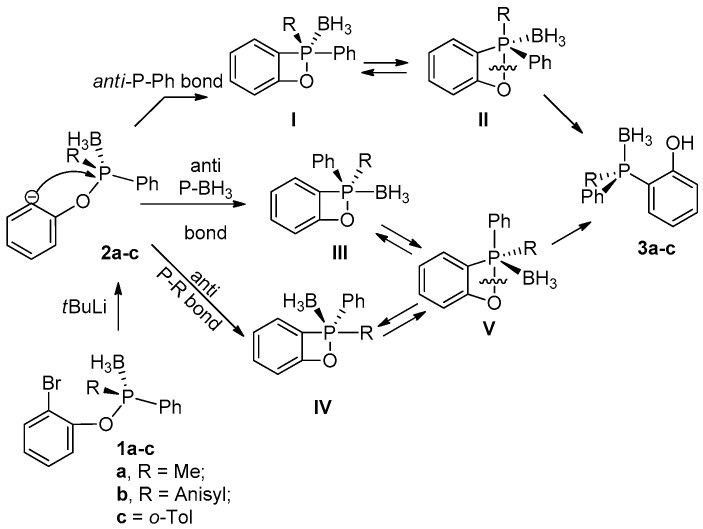
The rearrangement of the lithium salts **2a**–**c** derived from the 2-bromophenylphosphinite boranes **1a**–**c** to the *o-*hydroxyphenylphosphine boranes **3a**–**c**.

The participation of such chiral phosphoranes **I**–**V** as intermediates and their pseudorotation was suggested by Juge and coworkers [47] to explain retention of configuration during the rearrangement of the lithium salts **2a**–**c** derived from the 2-bromophenylphosphinite boranes **1a**–**c** to the *o-*hydroxyphenylphosphine boranes **3a**–**c** induced by halogen–metal exchange (Scheme 4) [47].

**Scheme 5:**
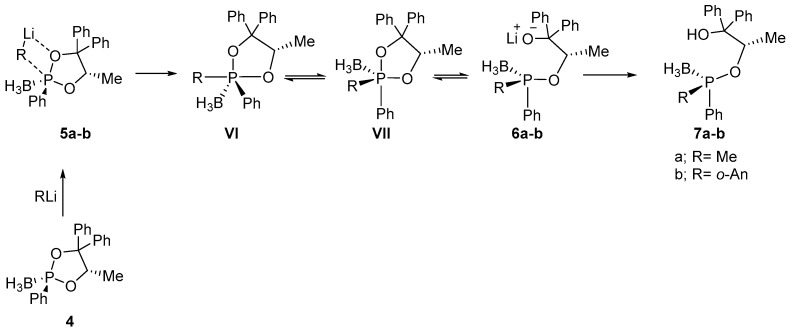
Stereochemical outcome of the regiospecific ring opening reaction of the diastereomerically pure dioxaphospholane borane **4** with organolithium reagents.

Earlier the same group proposed that pseudorotation of the intermediate phosphoranes **VI**, **VII** was responsible for predominant retention of configuration during the regiospecific ring opening reaction of the diastereomerically pure dioxaphospholane borane **4** with organolithium reagents which afforded phosphinite boranes **7a**,**b** (Scheme 5) [48].

**Scheme 6:**
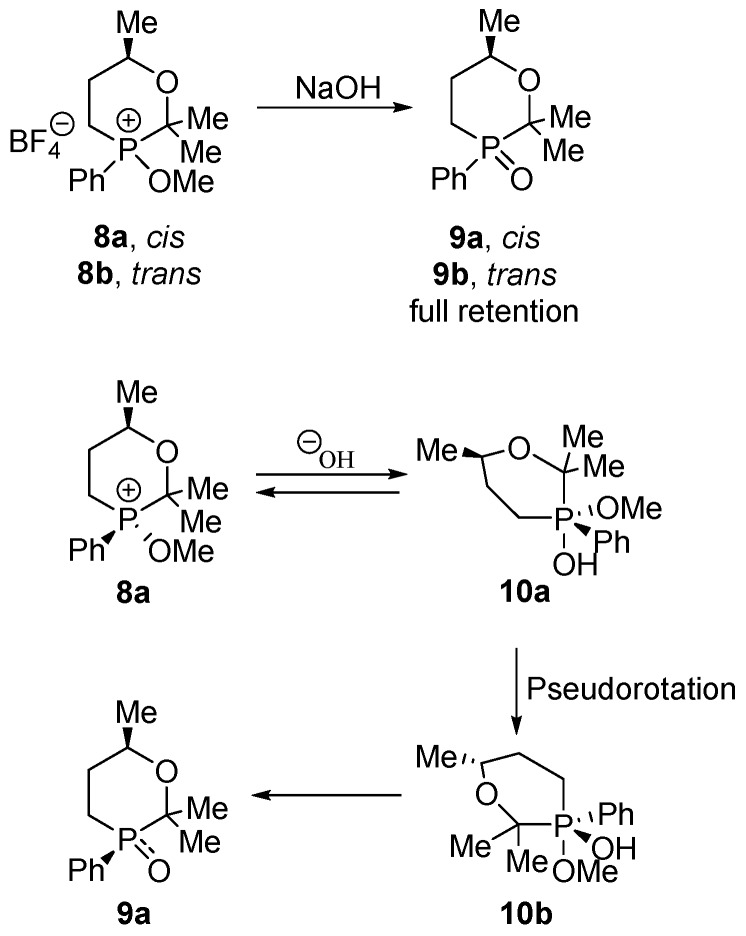
The hydrolysis of quasiphosphonium salts **8a** and **8b** involving two hydroxyphosphorane intermediates **10a** and **10b**.

As another example of such a transformation, hydrolysis of quasiphosphonium salts, *cis*- and *trans*-3-methoxy-2,2,6-trimethyl-3-phenyl-1,3-oxaphosphorinanium tetrafluoroborate salts **8a** and **8b** can be presented, by which the corresponding phosphine oxides **9a** and **9b** were formed with complete retention of configuration at phosphorus. The detailed NMR and stereochemical studies allow rationalization of the stereochemical outcome of this reaction in terms of the addition-elimination mechanism, A-E, involving two hydroxyphosphorane intermediates **10a** and **10b** (that are able to undergo pseudorotation) in which at least one oxygen is apical as shown in Scheme 6 [49].

**Scheme 7:**
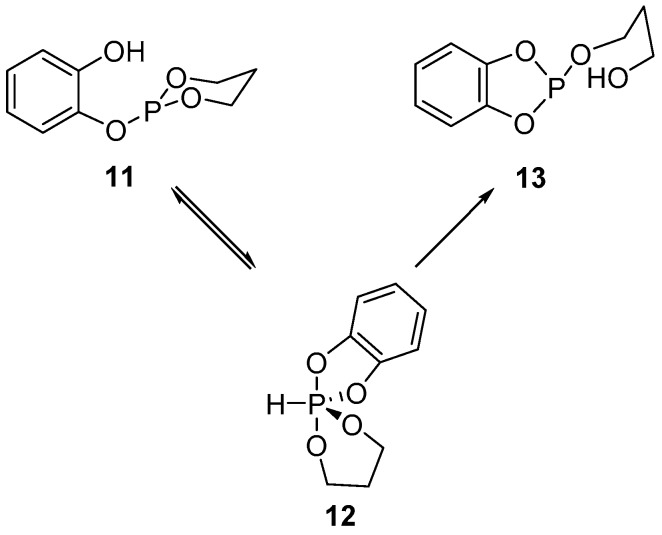
The intramolecular transesterification of the 2-hydroxyphenyl phosphite **11**.

Similarly, the participation of the chiral hydridophosphorane **12** as an intermediate in the intramolecular transesterification of the 2-hydroxyphenyl phosphite **11** to the 3-hydroxypropyl phosphite **13** (Scheme 7) was supported by low temperature ^31^P-NMR measurements [50].

**Scheme 8:**
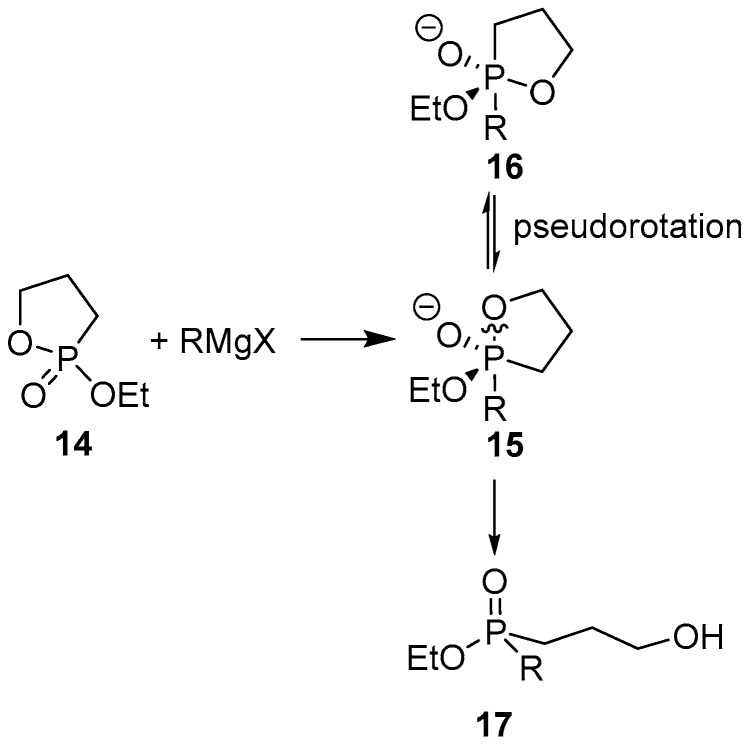
Reaction of cyclic oxaphospholane **14** and Grignard reagents.

Earlier, the participation of chiral hydridophosphoranes was proposed in the intermolecular transesterification of chiral asymmetric phenyl phosphites [51] and in the addition of achiral alcohols to compounds having a stereogenic tricoordinated phosphorus atom [52] and very recently in the hydrolysis of a trinucleoside monophosphate by the intramolecular 2α-hydroxy group neighbouring the scissile phosphodiester linkage [53]. The regioselective formation of chiral 3-hydroxypropylphosphinates **17** from cyclic oxaphospholane **14** and Grignard reagents was explained by assuming that during the reactions a pentavalent TBP phosphorane intermediate **15** is formed, which could not undergo pseudorotation to generate another phosphorane **16** due to a high energetic barrier. Therefore, the endocyclic P–O bond in **15** is cleaved almost exclusively to form the phosphinates **17** in high yields (Scheme 8) [54].

### 2.2. Chiral Phosphoranes and Their Anions as Isolable Species

As it was mentioned above, from the synthetic point of view, hypervalent structures exhibiting chirality due to the presence of one or two bidentate ligands should be more easily available than their acyclic analogues. Therefore, the majority of isolated hypervalent phosphoranes constitute mono- and especially bicylic derivatives in which a cyclic frame include pentacoordinated phosphorus atom bonded to a carbon and/or heteroatoms.

#### 2.2.1. Chiral Monocyclic Phosphoranes and Their Anions as Isolable Species

**Scheme 9:**
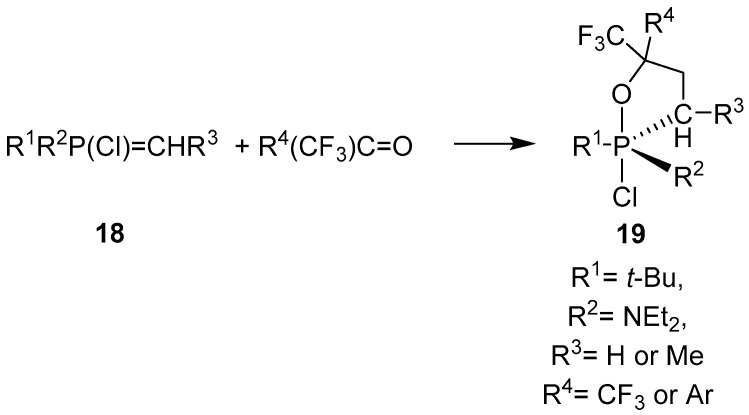
Monocyclic halophosphoranes **19**.

The non-concerted [2 + 2] cycloaddition reaction of P-haloylides **18** with trifluorometyl ketones led to the stereoselective formation of monocyclic halophosphoranes **19** (Scheme 9) [55].

**Scheme 10:**
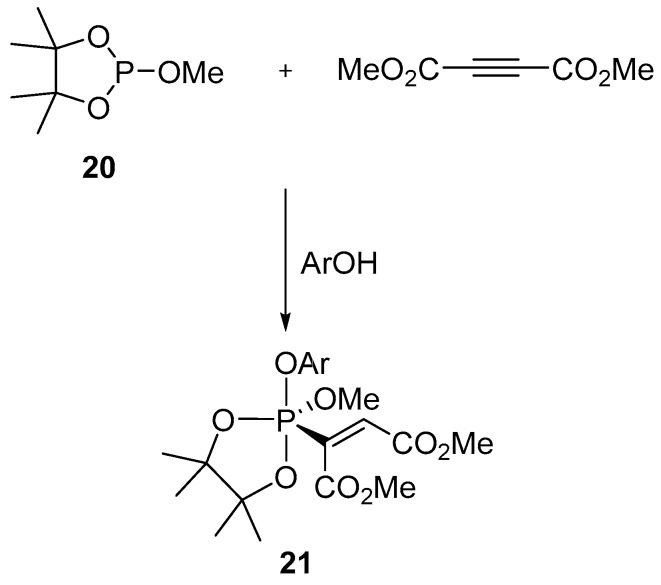
Reaction of cyclic phosphite **20** with dimethyl acetylenedicarboxylate.

The monocyclic vinyl phosphoranes **21** were prepared by the reaction of cyclic phosphite **20** with dimethyl acetylenedicarboxylate in the presence of the appropriate phenol (Scheme 10) [56].

**Scheme 11:**
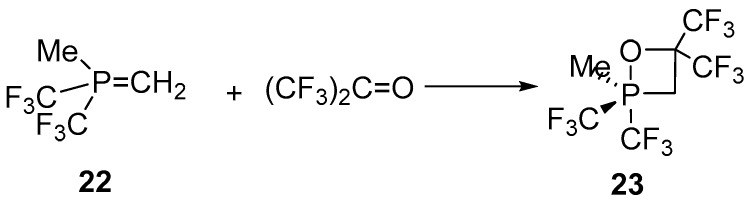
Reaction of ylide **22** with hexafluoroacetone.

The formation of monocyclic phosphorane **23** was observed in the reaction of ylide **22** with hexafluoroacetone (Scheme 11) [57].

**Scheme 12:**
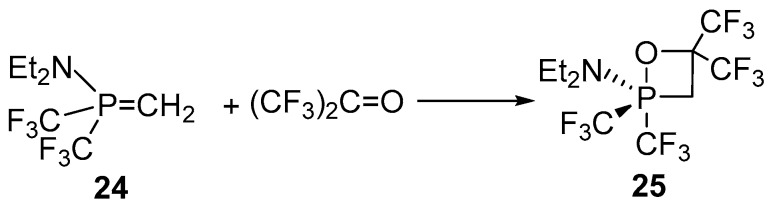
Reaction of ylide **24** with hexafluoroacetone.

A similar reaction of ylide **24** with hexafluoroacetone gave monocyclic phosphorene **25** (Scheme 12) [57].

**Scheme 13:**
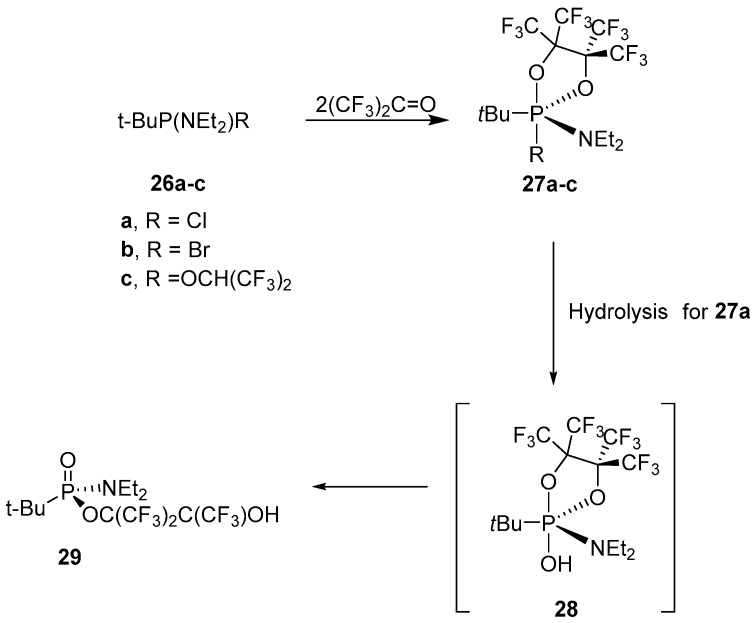
Reaction of organophosphorus derivatives **26a**–**c** with hexafluoroacetone.

The tricoordinate organophosphorus derivatives **26a**–**c** were found to react with hexafluoroacetone to give chiral monocyclic phosphoranes **27a**–**c** (Scheme 13) in which no ligand exchange processes were observed at room temperature. However, the chiral phosphonic acid ester **29** was isolated upon hydrolysis of the chlorophosphorane **27a**, which proceeds most probably via hydroxyphosphorane **28** (Scheme 13) [58].

**Scheme 14:**
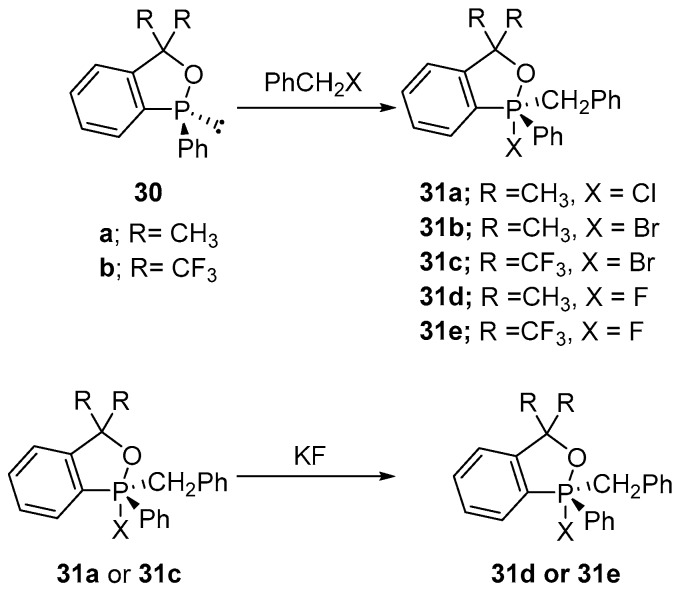
Preparation of monocyclic halophosphoranes **31a**–**e**.

Preparation of monocyclic halophosphoranes **31a**,**b** and **31c** was based on the reaction of the tricoordinate phosphinites **30a**,**b** with benzyl chloride or benzyl bromide. The corresponding fluorophosphoranes **31d** or **31e** were isolated upon the chloride-fluoride or bromide-fluoride exchange reactions when the chloride **31a** or bromide **31c** were used as a substrate respectively (Scheme 14) [59].

**Scheme 15:**
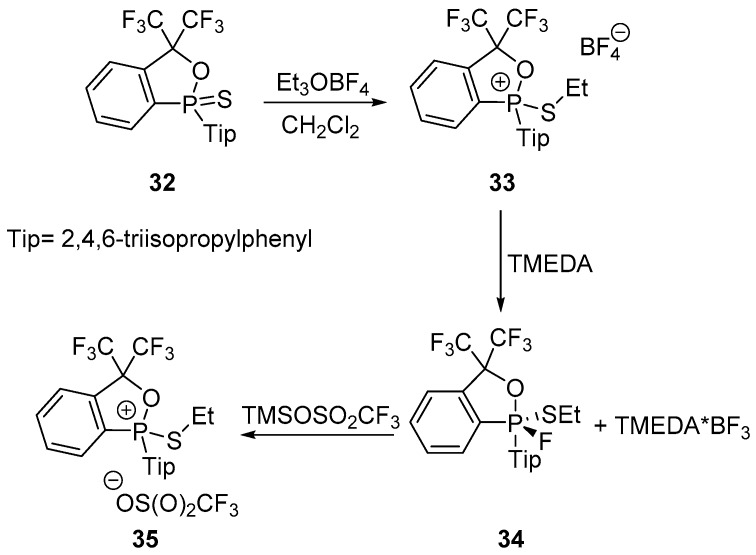
Preparation of monocyclic (ethylthio)fluorophosphorane **34**.

Monocyclic (ethylthio)fluorophosphorane **34** was isolated in quantitative yield upon fluoride anion abstraction from BF_4_ counter anion of phosphonium salt **33** easily generated by the reaction of cyclic phosphinothionate **32** with triethyloxonium tetrafluoroborate. Defluorination of the fluorophosphorane **34** with trimethylsilyl trifluoromethanesulfonate gave phosphonium salt **35** [60] (Scheme 15).

#### 2.2.2. Chiral Bicyclic Phosphoranes and Their Anions as Isolable Species

A relatively rich number of isolable bicyclic hypervalent phosphoranes have been well documented in the older and more recent chapters by Hellwinkel [61], Burger [62], Holmes [63], Husband and McNab [64], Burgada and Setton [65], Akiba [16], and Kawashima [66]. These chapters mention, more or less extensively, chiral bicyclic phosphoranes, but without deeper comments devoted to the phenomenon of chirality in such derivatives. Therefore, in this part of our compilation we are going to present, as comprehensively as possible, isolable hypervalent derivatives in which their chirality results from the presence of two heterocyclic units forming a P-spiro system. They are divided in accord with the commonly accepted Martin’s N-P-L (_n_A_m_B) coding system, in which N stands for the number of valence electrons associated formally with a central phosphorus atom and L shows the number of ligands (A and B stand for the bonding element) [14].

##### 10P-5C Phosphoranes

**Scheme 16:**
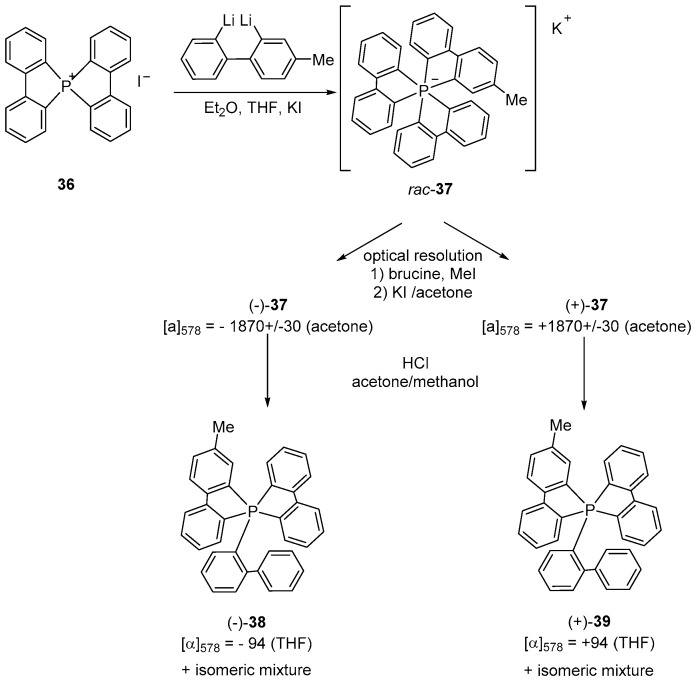
Preparation of enantiomeric spirophosphoranes **38**.

**Scheme 17:**
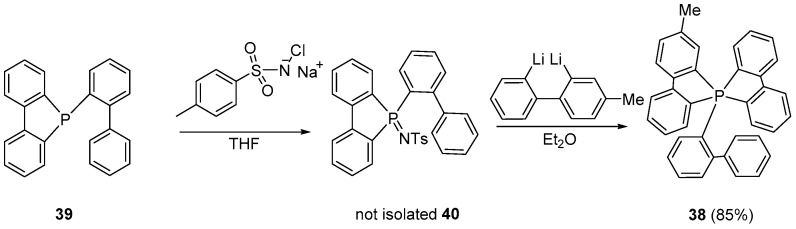
Preparation of **38** using biphenyl-biphenylyl-2-phosphine **39**.

Hellwinkel as early as 1966 reported on the isolation of the first optically active pentaarylphosphorane. Initially, hexacoordinate phosphorane potassium salt **37** was synthesized in good yield in racemic form by the reaction of bis(biphenyl)phosphonium iodide **36** with 2,2′-dilithio-4-methyl-biphenyl. The optical resolution of the racemic salt **37** was based on its conversion into two diastereomeric ammonium salts by the treatment with *N*-methylammonium iodide derived from brucine. The isolation of the levorotatory diastereoisomer ([α]_578_ = −1200) was achieved in moderate yield by recrystallization of a crude crystalline fraction from acetone. The dextrorotatory diastereoisomer of the ammonium salt was isolated from a mother liquor in poor yield and with a lower optical purity ([α]_578_ = +986). The isolated diastereoisomers were reconverted into the enantiomeric potassium salts upon the treatment with potassium iodide in acetone followed by repeating crystallizations. Their maximum specific rotation reached values [α]_578_ = + and −1870. Acidification of optically active potassium salts of hexacoordinate phosphorus derivatives (+) and (−)-**37** with HCl in methanol/acetone solution provided the mixture of spirophosphoranes from which enantiomeric spirophosphoranes **38** having [α]_578_ = + and −94, respectively were isolated by multiple recrystallization (Scheme 16). Additionally, the structure of **38** as a racemate was confirmed by the alternative method for its preparation using biphenyl-biphenylyl-2-phosphine **39** as the starting material (Scheme 17) [67].

**Scheme 18:**
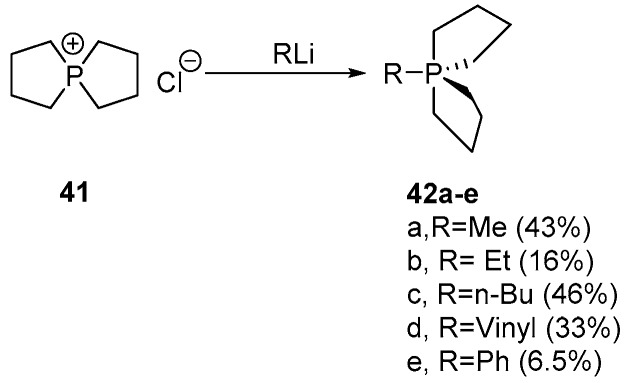
Reaction of spirocyclotetraalkylphosphonium salts **41** with a series of organolithium reagents.

More recently the reaction of spirocyclotetraalkylphosphonium salts **41** with a series of organolithium reagents RLi, was found to give spirobicyclophosphoranes **42a**–**e** in good to low yields (Scheme 18). Single crystal X-ray analysis of **42a** showed a TBP geometry with the axial-equatorial rings and the methyl group in an equatorial position. Low temperature NMR measurements indicated that all the pentacoordinate structures **42** show fluxional behaviour in a solution with a very low pseudorotation barrier [68].

##### 10P-4C-1O Phosphoranes

**Scheme 19:**
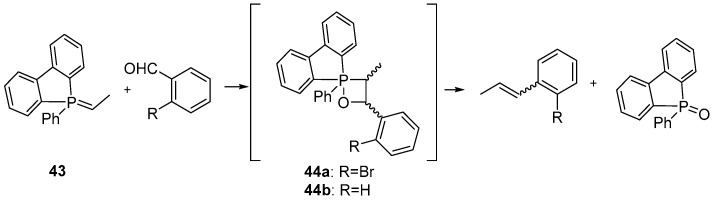
Reaction of biphenylphenylethylidene-phosphonium ylide **43** with benzaldehydes.

Oxaphosphetane (OPA) is a widely recognized intermediate formed via an irreversible [2 + 2] cycloaddition of an aldehyde or ketone with the phosphonium ylide in the Wittig reactions. The stereoselectivity of the reaction is decided by the course of the first irreversible step. Then, the oxaphosphetane undergoes irreversible [2 + 2] *syn* cycloreversion to give an alkene and phosphine oxide. In most cases the diastereomeric ratio of the oxaphosphetane (OPA) intermediate exactly corresponds to the final alkene, except the cases when a stereochemical drift occurs in the formation of the alkene product. The process “stereochemical drift” refers to the nonstereospecific decomposition of the oxaphosphetane (OPA) intermediate in reactions of certain alkylides with certain aldehydes. The particular investigations on the stereochemical drift in the Wittig reactions (alkylidenephosphonium ylide with aldehydes) under Li-salt-free conditions were undertaken by D. G. Gilheany et al. [69], and based on Variable-Temperature NMR measurements. A particularly suitable candidate for the study of stereochemical drift was intermediate **44a** formed in the reaction of biphenylphenylethylidene-phosphonium ylide **43** with 2-bromobenzaldehyde. It is due to its stability at room temperature (Scheme 19). It undergoes significant stereochemical drift on decomposition in THF at reflux: A sample containing *cis*- and *trans*-**44a** in a ratio of 94:6 gave alkenes of a (*Z*)/(*E*) ratio 82:18 under reflux conditions. On the other hand, the decomposition conducted at 50 °C was not complete after 6d, but the maximum possible (*Z*)/(*E*) ratio that could have been achieved in this reaction was 88:12, revealing that a smaller amount of the stereochemical drift had occurred. A very similar experiment supported with VTNMR spectra analysis over the temperature range −20 to +40 °C on **44b** (produced in the reaction of biphenylphenylethylidenephosphonium ylide **43** and benzaldehyde) was performed. Oxaphosphetane intermediate **44b** was formed in a diastereomeric ratio 71:29 (*cis*/*trans*). Again, the ratio of OPA, ylide and phosphine oxide was invariant within the temperature range from −20 to 30 °C, which indicates that no decomposition to alkene and phosphine oxide occurred. On heating the OPA **44b** was decomposed to produce the alkene in 53:47 (*Z*)/(*E*) isomeric ratio. In the ^31^P NMR spectra recorded for **44a** and **44b** in the presence of an excess aldehyde additional signals appeared in the pentavalent region (δ = −50 to −80 ppm). The observation was consistent with that reported earlier by Vedejs et al. [70]. Thus, the apparent interaction between OPA and aldehyde was expected to have an impact on the occurrence of the stereochemical drift.

##### 10P-3C-1N-1O Phosphoranes

**Scheme 20:**
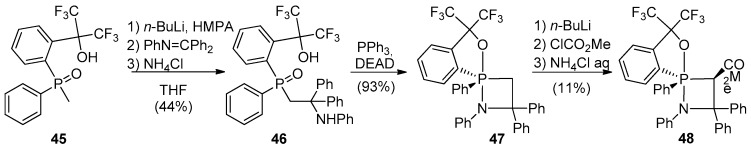
Synthesis of 1,2-Azaphosphetidine **47** and **48**.

In 1996 Kawashima and Okazaki succeeded in preparing pentacoordinate *N*-apical 1,2-azaphosphetidines, whose structures were fully characterized. They also investigated their thermolysis. The first observation on the occurrence of the *N*-equatorial pseudorotamers was also reported [71]. The synthetic pathway was as follows: the treatment of 2-(methylphenylphosphinyl)-α,α-bis(trifluoromethyl)phenylmethanol **45** with *n*-BuLi led to the generation of methylene carbanion species, which subsequently underwent nucleophilic addition to a Schiff base providing the β-aminoethyl derivative **46**. 1,2-Azaphosphetidine **47** was formed in 93% yield by intramolecular cyclization followed by dehydration under the Mitsunobu reaction condition (Scheme 20).

**Scheme 21:**
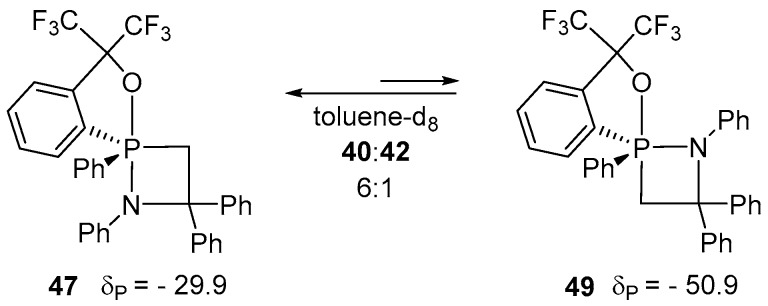
The equilibrium between phosphoranes **47** and **49**.

A typical chemical shift in the NMR spectra was observed for compounds with a TBP geometry in which an apical position was occupied with O or N atom. X-ray single crystal analysis revealed the distorted TBP geometry with phosphorus as a central core. Hydrolysis of **47** on silica gel resulted in its cycloreversion to the starting material **46**. Finally, 1,2-azaphosphetidine **47** was converted into the 3-methoxycarbonyl derivative **48**, likewise it had been reported, for the corresponding 1,2-λ^5^-oxaphosphetanes [72,73]. In the ^31^P-NMR spectrum recorded for the solution of **47** in deuterated toluene, apart from the appropriate resonance signal (δ_P_ = −29.9), another upfielded peak (δ_P_ = −50.9), was observed which was assigned to the *N*-equatorial pseudorotamer **49** (Scheme 21). A similar pseudorotamer (δ_P_ = −51) was observed also in the case of **48**.

**Scheme 22:**
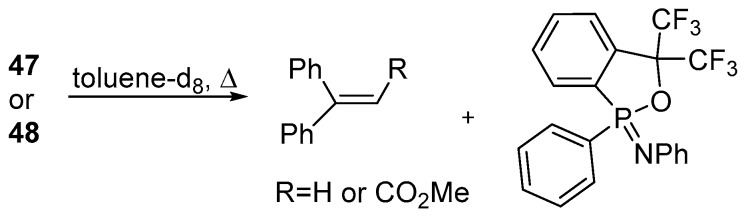
Thermolysis of **47** or **48**.

Thermolysis of **47** or **48** proceeded with the quantitative formation of the corresponding alkenes and iminophosphorane (Scheme 22), which implies that azaphosphetidines should be considered as aza-Wittig intermediates.

**Scheme 23:**
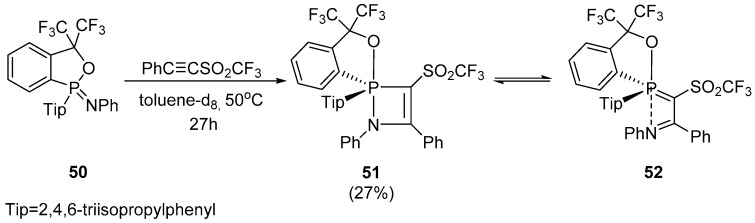
Cycloaddition reaction of the iminophosphorane **50** with an alkyne.

The reaction of an iminophosphorane, the nitrogen analogue of a phosphonium ylide, with a reactive alkyne has been intensely studied because it constitutes a useful synthetic pathway for the corresponding α-iminoalkylidenephosphoranes via a [2 + 2]-cycloadduct. The intermediate had neither been observed nor isolated until the first stable pentacoordinate l,2-λ^5^-azaphosphetine **51** was synthesized by Kawashima et al. [74]. Its preparation was achieved by the cycloaddition reaction of the Martin ligand-based iminophosphorane **50** with an alkyne (Scheme 23). X-ray crystallography of **51** showed a distorted TBP with N and O atoms at the apical positions. The variable temperature ^31^P NMR spectra of **51** in C_7_D_8_ or CD_3_CN showed a shift to a lower field with decreasing temperature indicating that (**51**) was in an equilibrium with the corresponding ylide structure **52**.

**Scheme 24:**
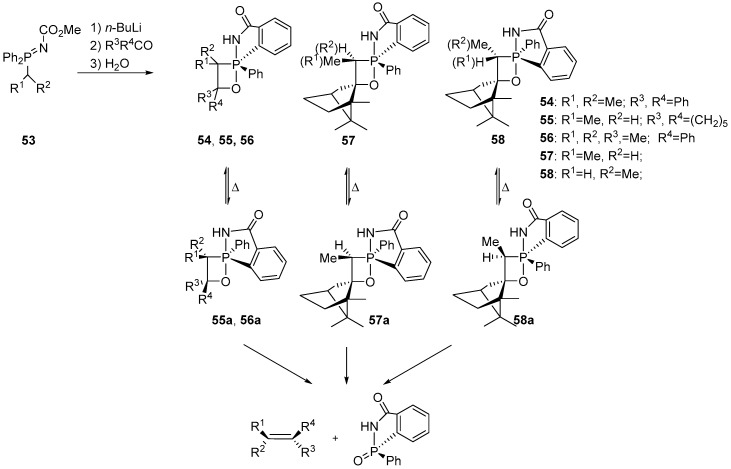
Synthesis of spiro-oxaphosphetanes **54**–**58** and their thermal decompositions.

The isomerization and thermal decomposition of isolable spiro-1,2-oxaphosphetanes has been studied in details with the aim to recognize a stereomutation mechanism of spiro-1,2-oxaphosphetanes, the Wittig intermediate products. Spiro-oxaphosphetanes **54**–**58**, stabilized by the presence of the *o*-benzamide moiety, were found to be good candidates for the studies [75]. The OPA’s **54**–**56** were obtained according to the previously reported procedure [76] (Scheme 24). The reactions with the hindered ketone, l-(−)-camphor, gave products **57** and **58** (in a 45:55 ratio) arising from the endo attack at the CO group of l-camphor.

All thermal decompositions of OPAs **54**–**58** furnished the appropriate alkenes and a phosphinamide, quantitatively. On the thermolysis of **55**–**58**, partial isomerization to **55a**–**58a** was observed, which resulted in the inverted configuration at phosphorus atom. It has been found that oxaphosphetane decomposition took place in a single step via a polar transition state. For the first time the stereomutation through three possible mechanisms MB_2_, MB_3_, and MB_4_ involving two, three, and four Berry pseudorotations (at phosphorus atom), respectively, supported by DFT calculations has been evidenced [75].

##### 10P-3C-2O Phosphoranes

**Scheme 25:**
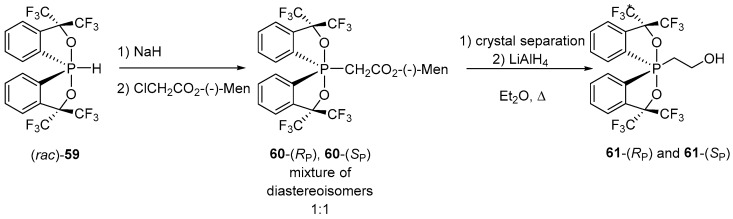
Synthesis of diastereomeric phosphoranes **60** and enantiomeric phosphoranes **61**.

The first configurationally stable enantiomeric pair of 10-P-3C-2O phosphoranes with well-defined sole stereogenic centre at phosphorus atom was successfully obtained and characterized by Akiba et al. [77]. The synthesis of diastereomeric **60**-(*R*_P_) and **60**-(*S*_P_) was achieved via a facile alkylation of the in situ generated phosphoranide anion by (−)-menthyl chloroacetate in 87% yield (Scheme 25). The diastereomeric mixture could be resolved effectively by fractional crystallization to furnish **60**-(*R*_P_) in 25% and **60**-(*S*_P_) in 22% yield. Thus, the separation and the selection of suitable crystals allowed for the determination of absolute configurations for both diastereoisomers by X-ray diffraction. The alkylation of P–H phosphorane derivatives was recognized to proceed with complete retention of configuration at the phosphorus sterereogenic centre, most probably due to no detectable racemization at the step of phosphoranide intermediate formation. 

**Scheme 26:**
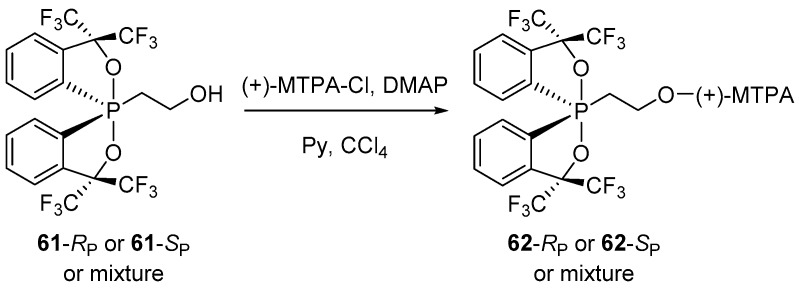
Conversion of phosphoranes **61** to esters **62**.

The removal of the menthyl unit by treating the diastereoisomers **60**-(*R*_P_) and **60**-(*S*_P_) with an excess of LiAlH_4_ provided the enantiomerically pure phosphoranes **61**-(*R*_P_) and **61**-(*S*_P_), respectively, with a single chirality center at the pentacoordinated phosphorus atom. In order to verify enantiomeric purity of the obtained *P*-chiral 2-hydroxyethylphosphoranes **61**, they were converted into the (*R*)-(+) Mosher esters **62** using (*R*)-(+)-2-methoxy-2-(trifluoromethyl)phenylacetic acid chloride ((+)-MTPA-Cl)) (Scheme 26) [78].

**Scheme 27:**
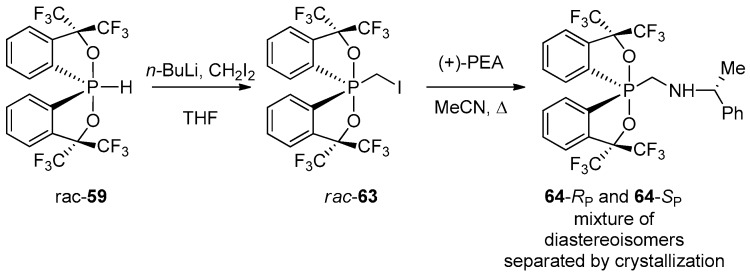
Synthesis of diastereomeric phosphoranes **64**.

Independently, the H-phosphorane **59** was converted (by the treatment with *n*-BuLi) into an anion, which was reacted with CH_2_I_2_ affording the alkylating product **63** in 82% yield. Subsequently α-iodomethyl phosphorane derivative was treated with α-methylbenzylamine to give a diastereomeric mixture of **64** in 56% yield (Scheme 27). The pure diastereoisomers were isolated by recrystallization and their absolute configuration was determined by X-ray analysis.

**Scheme 28:**
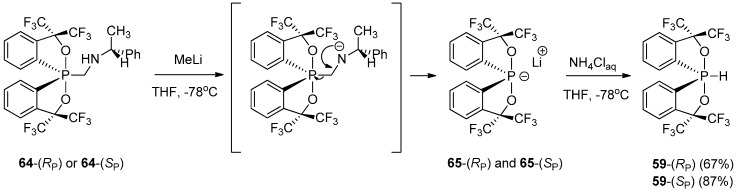
Conversion of diastereomeric phosphoranes **64** to enantiomeric phosphoranes **59**.

It was also found that deprotonation of the nitrogen-bonded proton in each single diastereomer **64** afforded enantiomeric phosphoroanidates **65**-(*R*_P_) and **65**-(*S*_P_) (via imine elimination). They, after acidification, gave the desired optically active P-H phosphoranes **59**-(*R*_P_) and **59**-(*S*_P_) (Scheme 28).

**Scheme 29:**
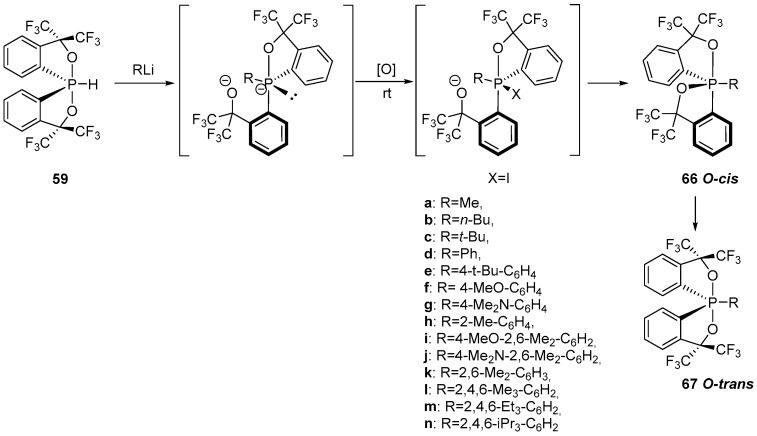
Synthesis of (*O-cis)* spirophosphoranes **66** and their stereomutation to (*O-trans*) phosphoranes **67**.

Later on, Akiba and co-workers reported the first example of an “anti-apicophilic” (*O-cis*) spirophosphoranes **67** [79,80,81], in which the oxygen atom occupies an equatorial position and the carbon atom is located in an apical position of a five membered ring, which is in contrast to the general concept of apicophilicity. According to the procedure shown in Scheme 29 (*O-cis*) phosphoranes **66** were obtained. The treatment of P–H phosphorane **59** with an excess of alkyl(aryl)lithium resulted in the formation of a dianionic intermediate which upon treatment with I_2,_ as an oxidizing agent, afforded P–I equatorial phosphorane. Its cyclization, initiated by the nucleophilic attack of the alkoxide anion at the phosphorous centre and the simultaneous extrusion of I^−^ produced *O-cis*-alkyl-or aryl spirophosphoranes **66**.

**Scheme 30:**
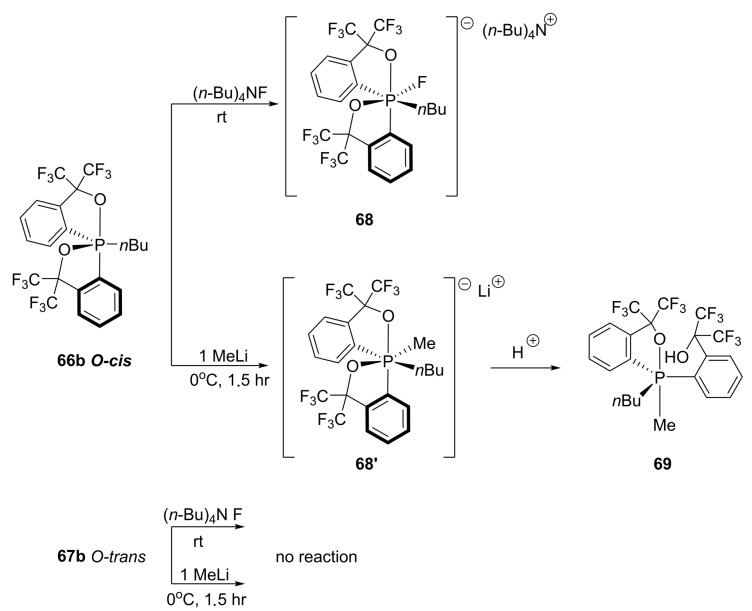
Reactivity of *O-cis*-**66b** and *O-trans*-**67b** toward nucleophiles.

The effects of substituents in the monodentate aryl ring of *O*-*cis* arylphosphoranes on the stereomutation process, along with a kinetic study on the stereomutation of *O-cis* **66k**–**n** to *O-trans* **67k**–**n** were studied in details. The exclusive generation of *O*-*cis* **66** could be observed for the alkyl derivatives **66a**–**c** right after oxidation, however, in the case of the phenyl derivative **66d** the *O-cis* isomer was the minor product. The pseudorotation to *O-trans* **67d** isomer was complete in 30 min at room temperature, which could be explained by the higher apicophilicity of aryl groups in comparison to alkyl groups. For compounds **66e**–**g**, bearing electron donating groups (*t*Bu, OMe, NMe_2_, respectively) in the 4-position of the monodentate aryl groups *O*-*cis* isomers were the major products. Finally, the use of bulky aryl organometallic reagents containing two substituents in *ortho* positions led to the exclusive formation of the *O-cis* isomers (**66i**–**n**), having the sufficient lifetimes for characterization. From the relative stability of **66i**–**n**, it is evident that steric effects contributed mainly to the stabilization against pseudorotation in these isomers that exhibited reversed apicophilicity. In the case of **66n**, pseudorotation was slow enough to allow for its isolation (purification on silica gel in 71% yield) and the crystal structure determination by X-ray diffraction. The differences in the reactivity of *O-cis*-**66b** and *O-trans*-**67b** toward nucleophiles were also indicated (Scheme 30). The reaction of *O-cis***-66** with TBAF as a nucleophile afforded the hexacoordinated phosphate **68** with the newly formed P–F bond while the *trans*-isomer did not react. Rapid decomposition of **68** caused by the presence of traces of water was observed. *O*-*Cis* **66b** likewise was reacted with MeLi to give product **69**, while *O*-*trans*-**67b** treated with organolithium reagent remained unreacted under the same conditions; however by increasing MeLi molar equivalency and temperature the product **69** was successfully isolated. 

**Scheme 31:**
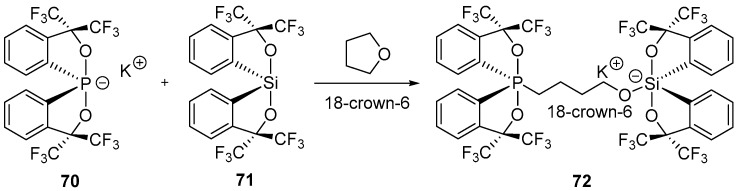
Reaction of phosphoranide **70** with silane **71**.

A three-component reaction of phosphoranide **70**, silane **71**, and THF was applied for the preparation of hypervalent compound **72** containing simultaneously pentacoordinated phosphorus and silicon atoms (Scheme 31) [82,83]. The ^31^P-NMR spectrum indicated the presence of two diastereomeric products in a ratio of 1:1.

**Scheme 32:**
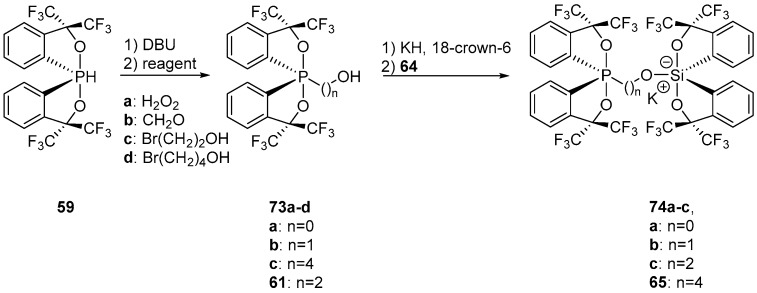
Reactivity of phosphoranes **59**, **61** and **73**.

The synthesis of other phosphoranylalkoxysilicates was achieved by the nucleophilic addition of phosphoranylalkoxides generated from hydroxy(alkyl)phosphoranes **61** and **73a**–**c** to silane **74** (Scheme 32). Hydroxy(alkyl)phosphoranes **61** and **73a**–**c** were prepared by deprotonation of hydrophosphorane **59** with DBU and subsequent treatment with an electrophile, such as hydrogen peroxide, formaldehyde, 2-bromoethanol, and 4-bromobutanol, respectively. Their treatment with KH in the presence of 18-crown-6 followed by the addition of silane **71** in THF afforded the appropriate phosphoranyloxysilicate **74a** (56%), and phosphoranylalkoxysilicates **74b**,**c** and **72** (53%–96%). Potassium silicates **74a**–**c** were obtained as a mixture of two diastereomers (in ratios of 66:34 for **74a**, 53:47 for **74b**, 50:50 for **74c**, respectively), as estimated by the integrals in the ^31^P-NMR spectra [82,83].

**Scheme 33:**
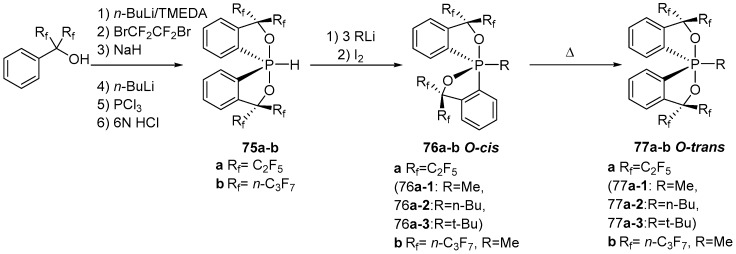
Synthesis of phosphoranes **76** and **77**.

More recently, studies on spirophosphoranes, prepared from a new bulkier analogue of the Martin ligand containing two decafluoroetyl units, have been carried out by the Yamamoto group. This new ligand was expected to suppress BPR more efficiently than the Martin ligand [84]. The synthesis of P–H-spirophosphoranes **75a**,**b** was based on a two-step reaction involving dimetallation of perfluorocumyl alcohol derivatives leading to the corresponding dianion and its subsequent condensation with PCl_3_, followed by acidification with 6 molar HCl. The *O*-equatorial spirophosphoranes **76a**,**b** were obtained by the reaction of **75a**,**b** with 3 molar equivalents of RLi followed by the oxidation with I_2_. Their conversion to the corresponding *O*-apical stereoisomers **70a**,**b** proceeded in the solution at high temperatures. The stereomutation of **76a-1** to **77a-1** was found to proceed with first–order kinetics. The free energy of activation for stereomutation was higher by 3.6 kcal/mol in comparison with the CF_3_ analogue indicating that steric hindrance of pentafluoroethyl group is the major factor for freezing pseudorotation. Recently, Jiang and coworkers [85] obtained a new stable anti-apicophilic phosphorene **76b** with four bulky *n*-C_3_F_7_ groups, which was converted to more stable *O*-apical phosphorane **77b** only by heating, (Scheme 33).

**Scheme 34:**
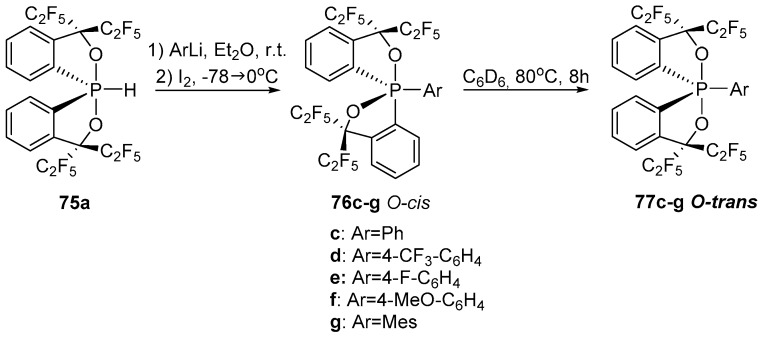
Synthesis of phosphoranes **76** and their interconversion into **77**.

The same research group elaborated the synthesis of a series of anti-apicophilic phosphoranes bearing a *para*-substituted aryl group (–C_6_H_4_(*p*-X); X = H, CF_3_, F, OMe) or a mesityl group as a more electronegative monodentate ligand than an alkyl group. The *O*-equatorial arylphosphoranes **69c**–**g** were successfully synthesized by the reaction of the P-H spirophosphorane **68a** with an excess of ArLi, followed by treatment with I_2_ (Scheme 34) [86]. These phosphoranes were found to be stable at r.t. Their isolation indicates that the steric effect of the C_2_F_5_ groups for freezing the isomerization is remarkable. By heating in an organic solvent the *O*-equatorial arylphosphoranes **69c**–**g** were quantitatively converted into the more stable *O*-apical isomers **70c**–**g**. The effect of a solvent and a para-substituent on the rate of the isomerization were also investigated in order to provide an insight into the π → σ* P–O interaction in the *O*-equatorial arylphosphoranes. The kinetic study showed a small para-substituent effect on the stereomutations. However, the multi-regression analysis for the para substituents revealed the 1.3 times greater contribution of the resonance effect on the on the isomerization rate in C_6_D_6_ than the inductive effect suggesting that the π → σ* P–O stabilizing interaction in the *O*-equatorial isomer plays some role in the isomerization.

**Scheme 35:**
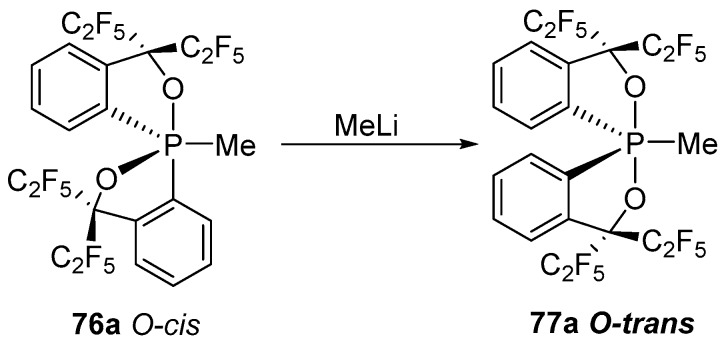
Methyllithium induced epimerization of *O*-equatorial methylphosphorane **76a**.

In this context it is interesting to note that the *O*-equatorial methylphosphorane **76a** treated with MeLi was converted to the more stable *O*-apical isomer **77a**, but on the contrary, the *O-*apical phosphorane did not react with MeLi at all (Scheme 35) [87].

**Scheme 36:**
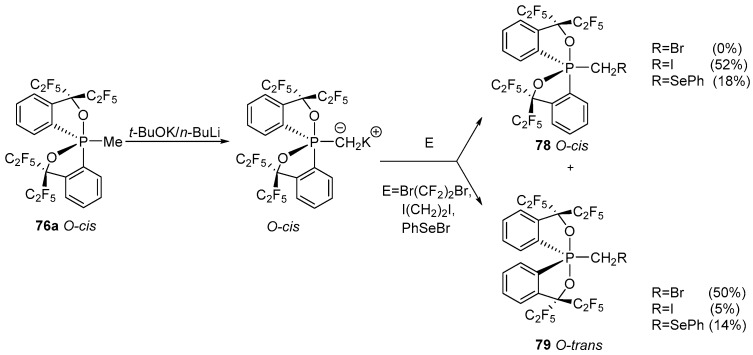
Synthesis of phosphorane derivatives **78** and/or **79**.

Deprotonation of methylphosphorane **76a** at the methyl group using superbase (*t*-BuOK/*n*-BuLi) led to the corresponding α-anion, which upon treatment with electrophilic agents gave new phosphorane derivatives **78** and/or **79** (Scheme 36). The *O*-equatorial and *O*-apical phosphoranes having a β-hydroxyethyl group were synthesized according to this procedure using paraformaldehyde and applied as monodentate ligands [87]. 

Akiba et al. reported the preparation and characterization of an anti-apicophilic spirophosphorane bearing an oxaphosphetane ring and the Martin ligand [88]. Although the substitution pattern on the phosphorus atom slightly differs from that of oxaphosphetane in the typical Wittig reaction they can be considered as a model for the possible reactive intermediate for the Wittig reaction.

**Scheme 37:**
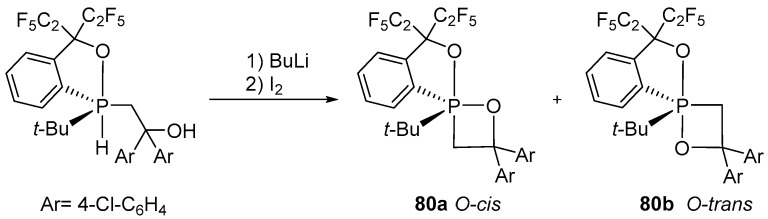
Synthesis of isomeric mixture of oxaphosphetane **80a** and **80b**.

The synthesis of isomeric mixture of oxaphosphetane **80a** and **80b** in 1:1 ratio was achieved by the oxidation procedure using *n*-BuLi followed by iodine in ether (Scheme 37). The sole *anti*-apicophilic **80a** isomer was isolated from the reaction mixture by crystallization upon addition of hexane. Under acidic conditions **80a** underwent complete stereomutation to **80b**. A similar conversion took place within minutes upon dissolution of **80a** in anhydrous CDCl_3_ at room temperature. A ^31^P-NMR measurement of the **80b** sample heated to 120 °C in *p*-xylene did not show the presence of **80a** in equilibrium, indicating that **80a** is thermodynamically much less favorable than its *O-*apical isomer. Moreover, heating a solid sample of **80a** at 120 °C for 5 min. gave only **80b** as a result of pseudorotation. 

**Scheme 38:**
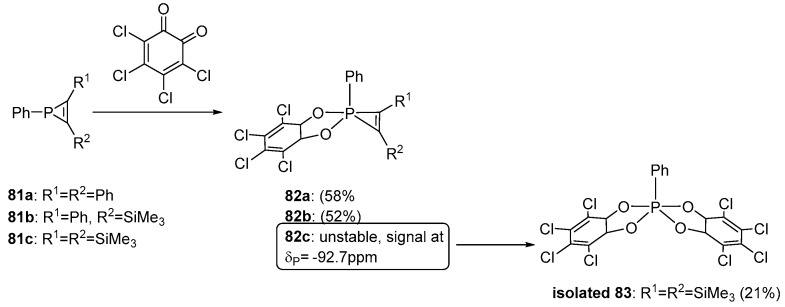
Reaction of phosphirenes **81a** and **81b** with *o*-chloranil.

The synthesis of pentacoordinate phosphirenes **82a** and **82b** bearing a phenyl group on the phosphorus atom was achieved by the reaction of tricoordinate phosphirenes **81a** and **81b** with *o*-chloranil (Scheme 38) [89]. 

The formation of **82c** was evidenced by ^31^P-NMR spectrum that showed a suitable signal at δ_P_ −92.7. It was converted rapidly to phosphorane **83** bearing two tetrachlorocatecholate ligands. For relatively more thermally stable phosphirene **82a** the crystal structure was determined by X-ray analysis. It showed that phosphirene **82a** has a highly distorted square pyramidal SP arrangement at the phosphorus atom as a core. The basal positions are occupied by two oxygen atoms from the tetrachlorocatecholate ligand and two carbon atoms belonging to the three-membered ring of phosphirene, when the phenyl group is located at the apical position. No decomposition was observed for **82a** after heating at 60 °C for 40 h. On the other hand, phosphirene **82b** was thermally less stable, and decomposed at room temperature to give the *o*-chloranil derived phenylphosphinite and phenyl(trimethylsilyl)acetylene. These results indicate that an attachment of a trimethylsilyl group to the endocyclic carbon lowers the thermal stability of the pentacoordinate phosphirenes.

##### 10P-3C-1O-1Se; 10P-3C-1O-1S; 10P-3C-1O-1F; 10P-3C-1O-1H Phosphoranes

**Scheme 39:**
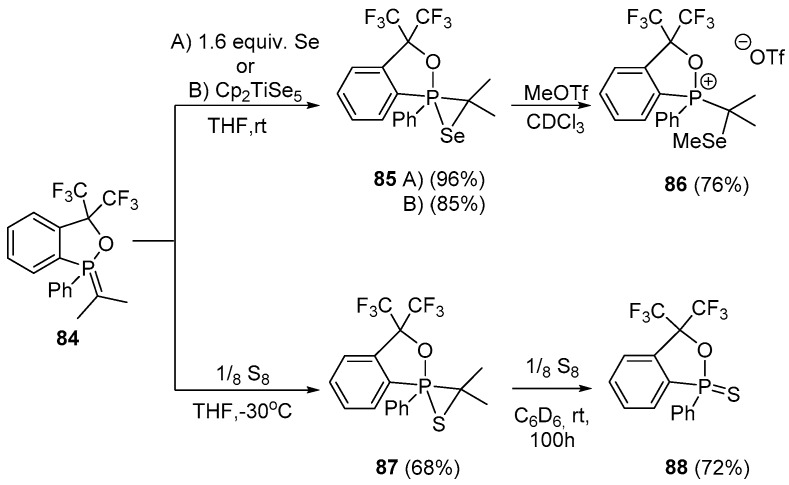
Reactions of monocyclic phosphorus ylide **84**.

The synthesis of the first 1,2-σ^5−^selenaphosphirane and 1,2-σ^5^-thiaphosphirane involving a pentacoordinate phosphorus atom was based on the reaction of the Martin ligand based phosphorus ylide with elemental selenium [90], CpTiSe_5_ [91] or elemental sulfur [92]. Treatment of ylide **84** with 1.6 equiv. of elemental selenium in THF at room temperature or with Cp_2_TiSe_5_ under the same conditions resulted in the formation of selenaphosphirane **85** as a solid in 96% and 85% yield, respectively (Scheme 37). Although the selenaphosphirane **85** is highly moisture-sensitive, its isolation was successful under an argon atmosphere and its structure was crystallographically characterized. Both, the solid-state ^31^P{^1^H} NMR spectrum and the ^31^P{^1^H} NMR spectrum of the C_6_D_6_ solution showed a single peak with chemical shifts at −26.1 and −26.6, respectively. This was considered as evidence for the pentacoordinate state of the phosphorus atom of the selenaphosphirane. It is interesting to note that in the ^77^Se{^1^H}-NMR spectrum (the resonance signal at δ_Se_ 147.5 in C_6_D_6_), the coupling constant between the phosphorus and the selenium nuclei was not observed. This selenaphosphirane upon treatment with methyl triflate in CDCl_3_ afforded the highly air-sensitive α-(methylseleno)-α-methylethyl phosphonium triflate **86** in 76% yield (Scheme 39). The formation of **86** was initiated by the electrophilic attack of methyl triflate on the negatively charged selenium atom in **85** and proceeded with the subsequent cleavage of the polarized P–Se bond. In a similar manner the thiaphosphirane **87** was prepared in 68% yield, by the reaction of the ylide **84** with 0.96 equivalent of elemental sulfur in THF at −30 °C for 7 h (Scheme 39). Thiaphosphirane **87** was found to be air-sensitive and its single crystal was obtained by recrystallization from hexane under an argon atmosphere. Its X-ray crystallographic analysis showed that the oxygen and sulfur atom are located at apical positions.

Thiaphosphirane **87** showed inertness toward triaryl- or trialkylphosphines. This is in a contrast to tri- and tetracoordinate thiaphosphiranes which underwent desulfurization to form phosphaalkene derivarives and phosphine sulfides [93]. When the thiaphosphirane **87** was treated with additional portion of elemental sulfur the corresponding phosphinothionate **88** was isolated in 72% yield. The independent experiment showed that the phosphorus ylide **84** treated with 2 equiv. of elemental sulfur provided phosphinothionate **88** in 31% yield.

##### 10P-2C-2N-1O; 10P-2C-1N-2O Phosphoranes

**Scheme 40:**
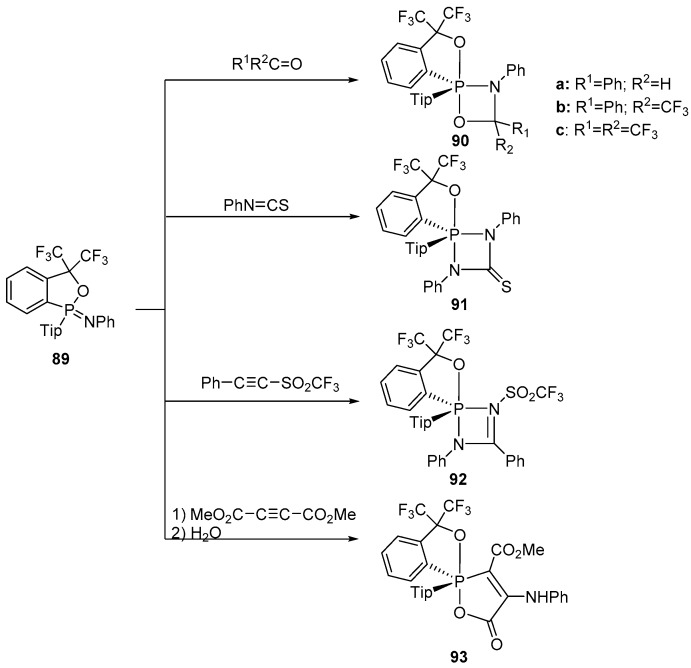
Syntheses of bicyclic phosphoranes **90**–**93**.

The reactions of iminophosphorane bearing the Martin ligand and a bulky 2,4,6-triisopropyl-phenyl group with a ketone, an isothiocyanate and an alkyne has been found to proceed with the formation of the corresponding cycloadducts as novel heterocycles bearing pentacoordinated phosphorus atom [94,95]. Cyclic iminophosphinate **89** reacted with carbonyl compounds, phenyl isothiocyanate and phenylethynyl trifluoromethyl sulfone affording the appropriate cycloadducts: 1,3,2λ^5^-oxazaphosphetidines **90a**–**c**, 1,3,2λ^5^-diazaphosphetidine-4-thione **91**, 1,2λ^5^-azaphosphetine **92**, respectively. The reaction with hexafluoroacetone proceeded at room temperature yielding cyclic phosphorane which could be even purified by silica gel chromatography. With dimethyl acetylenedicarboxylate (DMAD) and water the reaction provided 1,2λ^5^-oxaphosphol-5(2*H*)-one **93** instead of the expected 1,2-azaphosphetine (Scheme 40).

**Scheme 41:**
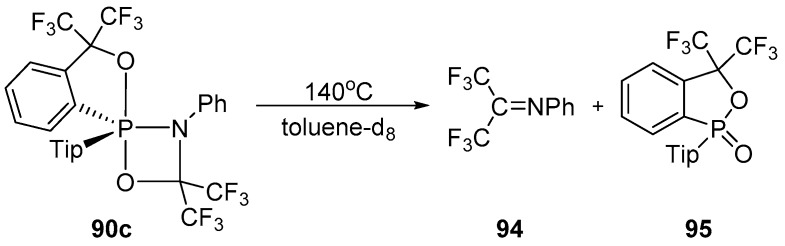
Thermolysis of spirophosphorane **90c**.

Thermolysis of **90c** at 140 °C in a sealed tube gave the corresponding imine **94** and cyclic phosphinate **95** (Scheme 41), indicating that 1,3,2λ^5^-oxazaphosphetidine **90c** is regarded as an intermediate of the aza-Wittig reaction [95].

**Scheme 42:**
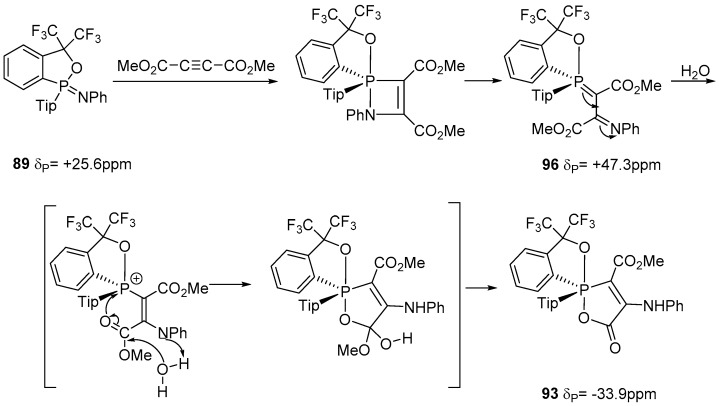
The mechanism of formation of the bicyclic phosphorane **93** from cyclic iminophosphinate **89**.

The detailed studies on the formation of 1,2λ^5^-oxaphosphol-5(2*H*)-one **93** were conducted on the basis of ^31^P-NMR analysis [96]. In the course of the reaction a decrease of the resonance signal at +25.6 ppm along with an increase of the new one at +47.3 ppm corresponding to **96** was observed. The latter was found to be unstable in the presence of moisture which resulted in the slow conversion to the final product. The proven synthetic pathway was concluded in Scheme 42.

##### 10P-2C-2O-1Z (Z = N, O, etc.) Phosphoranes

**Scheme 43:**
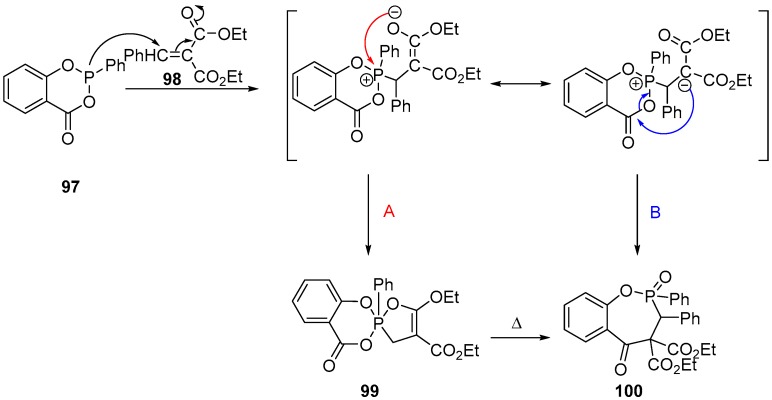
Synthesis of pentacoordinate phosphorane **99** and phosphinate derivative **100**.

It is already well known that the preparation of pentacoordinate phosphoranes can be accomplished by the reaction of phosphorus (III) compounds with ylidene derivatives of β-carbonyl compounds. This approach was exemplified by the reaction of 2-phenyl-1,3,2-benzodioxaphosphorin-4-one **97** with diethyl benzylidenemalonate **98** which occurred along two pathway, yielding pentacoordinate phosphorane **99** and phosphinate derivative **100** containing tetracoordinate phosphorus atom [97]. When the reaction was allowed to heat for a short time the formation of the seven-membered phosphonate derivative with relatively high stereoselectivity equal to 50% was observed (Scheme 43).

**Scheme 44:**
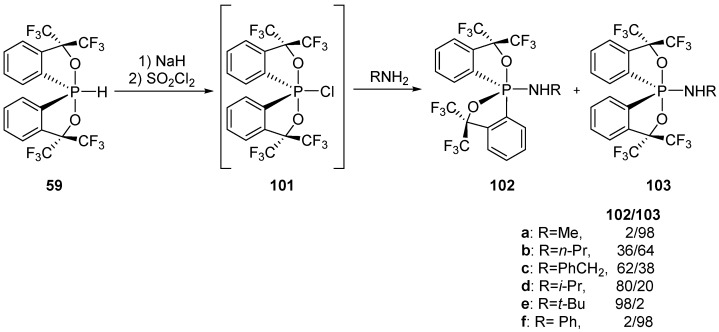
Synthesis of aminophosphoranes **102** and **103**.

The plausible mechanism involves the nucleophilic attack of the phosphorus atom at the β-carbon atom in α,β-unsaturated carbonyl compounds to produce an intermediate with delocalized π-electrons along three atoms, as is presented by its two resonance structures. In case of synthetic pathway A the attack of negatively charged oxygen on phosphorus atom occurs with reversible formation of pentacoordinate phosphorane. The second synthetic pathway (B) assumes nucleophilic substitution at the carbonyl carbon atom to yield the seven-membered ring, 1,2-benzoxaphosphepine. In further investigations Akiba et al. [98] succeeded in preparing spirophosphoranes bearing Martin ligand units and a monodentate primary amino substituent. These *O*-equatorial isomers were found to be surprisingly stable, taking into account the fact that the equatorial substituents, which are more electronegative than carbon, are expected to facilitate pseudorotation. The *O*-equatorial aminophosphoranes **102** along with *O*-apical isomers **103** were obtained from the in-situ generated chlorophosphorane **101** which was allowed to react with the corresponding amines (Scheme 44). However, when methylamine or aniline were used, the corresponding *O*-equatorial isomer were not produced.

The *O*-equatorial phosphoranes **102** were stable at room temperature and could still be converted to their more stable *O*-apical pseudorotamers **103** when they were heated in a solution, indicating that the phosphoranes **102** are kinetic products, as in the case of *O*-equatorial alkylphosphoranes. The structures of **102c** and **102e** were determined by single-crystal X-ray analyses to be in the *O*-equatorial configuration. The unusual stability of the *O*-equatorial phosphorane **102** could be attributed to the stabilizing n_N_ → σ*_P-O_ orbital interaction. More detailed studies on the experimental determination of the n_N_ → σ*_P-O_ interaction energy of *O*-equatorial *C*-apical phosphorane supported with DFT calculations have been also reported providing an additional proof for this assumption.

**Scheme 45:**
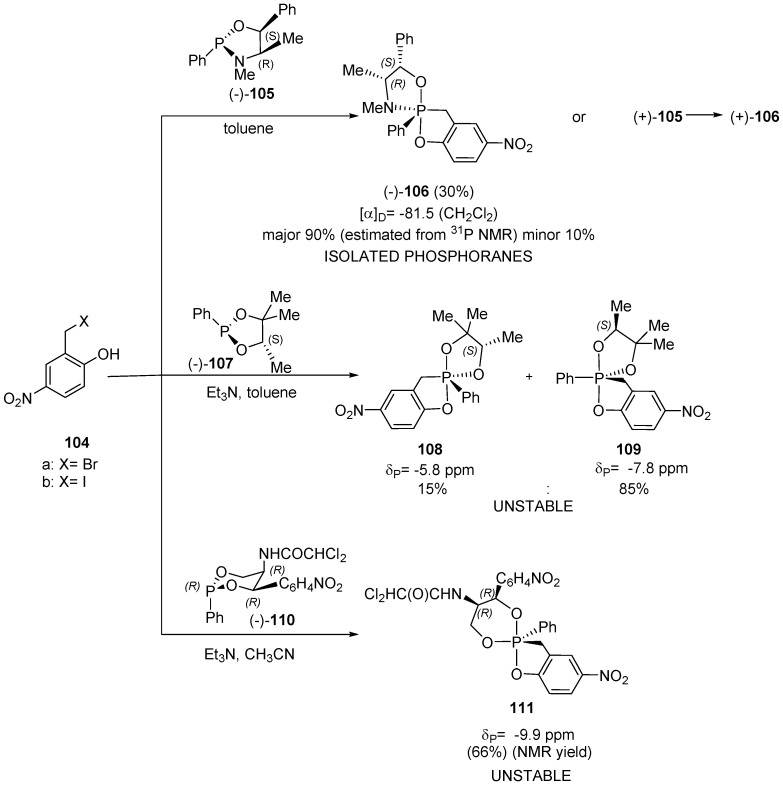
Reactions of cyclic five or six-membered tricoordinated phosphorus compounds with 2-hydroxy-5-nitrobenzyl halides.

Juge et al. described the formation of chiral bicyclic phosphoranes in the Arbuzov-type reaction of diastereomerically pure cyclic tricoordinated oxaphosphacycloalkanes and very reactive Koshland reagent I **104** [99]. They found that both enantiomers of 2-phenyl-1.3.2-oxazaphospholidine [(−)-**105** derived from (+)-ephedrine or (+)-**105** from (−)-ephedrine], treated with 2-hydroxy-5-nitrobenzyl bromide **104a** afforded the stable pentacoordinated phosphoranes [(−)-**106** from (−)-**105** or (+)-**106** from (+)-**105**] which were isolated by simple chromatography. The minor isomeric spirophosphoranes with the opposite configuration at the phosphorus atom were also formed (Scheme 45). 2-Hydroxy-5-nitrobenzyl halides **104a**,**b** reacted also with five- or six-membered cyclic tricoordinated phosphorus compounds (−)-**107** (derived from (+)-2-methyl-2,3-butanediol) or (−)-**110** (derived from (−)-chloramphenicol), yielding spirophosphoranes, **108** and **109** or **110**, respectively. However, these spirophosphoranes were too unstable to be isolated. Their formation was confirmed only by the ^31^P-NMR spectra of a crude reaction product.

##### 10P-1C-1N-3O Phosphoranes

**Scheme 46:**
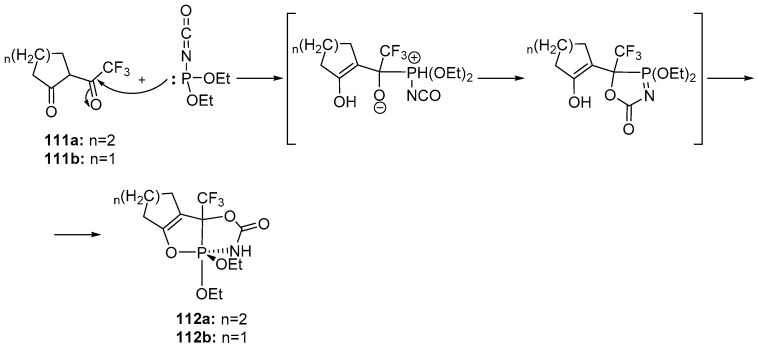
Synthesis of bicyclic aminophosphoranes **112**.

The synthesis of a pentacoordinated phosphorane containing tricyclic system was reported by Sevenard et al. [100]. 2-Fluoroacetylcycloalkanones **111a**,**b** reacted with diethyl isocyanatophosphite diastereospecifically leading to phosphoranes **112a**,**b** via intermediate species formed by the addition of phosphorus to a trifluoromethyl substituted carbonyl atom which subsequently underwent two additional heterocyclizations (Scheme 46).

The phosphorane derived from 2-trifluoroacetylcyclopentanone **112b** was moisture-sensitive, unlike the cyclohexane derivative **112a**, probably due to the greater ring strains. The proposed structure was strictly confirmed by means of NMR data on the basis of which it was found that carbon atom occupies an axial position and two annulated five membered rings are arranged in the equatorial-axial-equatorial order.

##### 10P-1C-2N-2O Phosphoranes

**Scheme 47:**
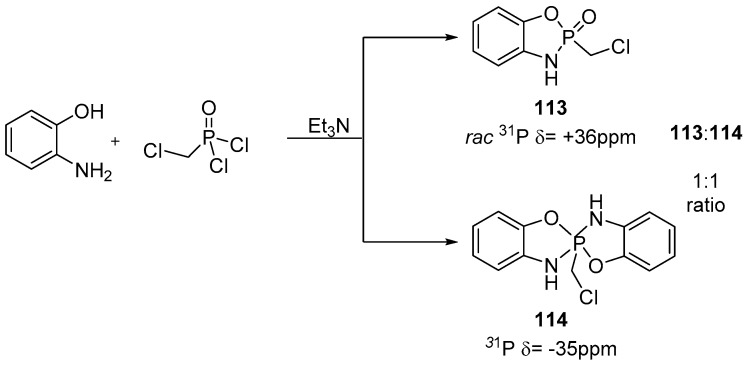
Synthesis of bicyclic aminophosphorane **114**.

Pudovik et al. reported the synthesis and crystal structure analysis of spirophosphoranes derived from benzoxazaphospholidines and/or 2-aminophenol [101]. It has been found that the condensation reaction of 2-aminophenol with chloromethylphosphonic dichloride proceeded with the formation of the expected benzoxazaphospholidine-2-oxide **99** along with the unexpected one: spirophosphorane **100** (Scheme 47).

**Scheme 48:**
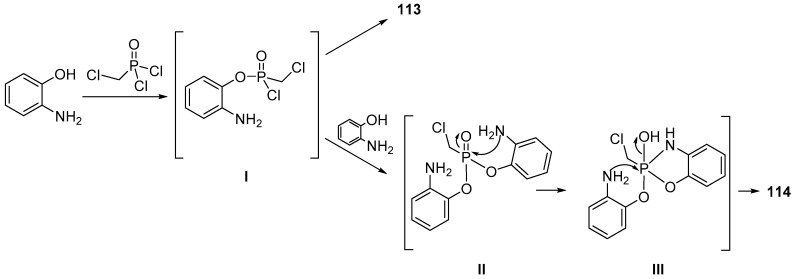
The mechanism for the formation of spirophosphorane **114**.

As indicated by the authors, the crucial step in a plausible pathway for their synthesis is an initial phosphorylation of a hydroxy group in 2-aminophenol with phosphonic dichloride leading to chloromethylphosphonochloridate intermediate (I). Subsequent nucleophilic attack of the amino group at the phosphorus atom leads to the cyclization product **99**. Simultaneously, the second pathway is realized when chloromethylphosphonochloridate intermediate reacts with the next molecule of the starting aminophenol to give the OH condensation product, which undergoes cyclization to provide unstable intermediate (II, III). It is then converted to the spirophosphorane as a result of intramolecular nucleophilic attack of the amino group at electrophilic phosphorus atom with the elimination of water (Scheme 48).

**Scheme 49:**
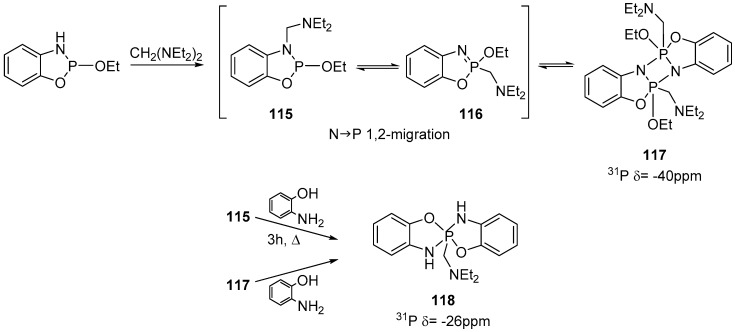
Syntheses of bicyclic diaminophosphoranes **117** and **118**.

The alkylation of 2-alkoxy-1,3,2-benzoxazaphospholidine with bis(dialkylamino)methanes afforded N-alkylated product **115** which underwent tautomerization to phosphonimidate **116** and further transformation to polycyclic phosphoranes **117** in 30 days [102]. It is worth to mention that both benzoxazaphospholidine **115** and polycyclic phosphorane **117** were used as phosphorylating agent for 2-aminophenol yielding spirophosphorane **118** (Scheme 49). The crystal and molecular structure of the synthesized spirophosphoranes **114** and **118** were determined by single-crystal X-ray diffraction [101,103,104].

**Scheme 50:**
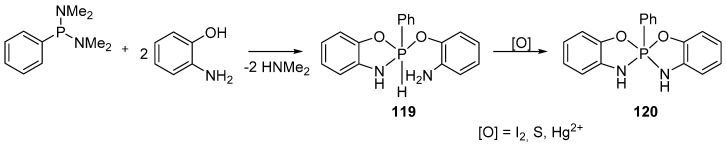
Synthesis of bicyclic diaminophosphorane **120**.

Amino alcohol based monocyclic hydrophosphorane **119** was prepared in the reaction of 2-aminophenol with tetraalkylphenylphosphonous diamide and used for the preparation of bicyclic spirophosphorane **120** [105] based on the oxidation procedure or on heating above its melting point (Scheme 50). 

**Scheme 51:**
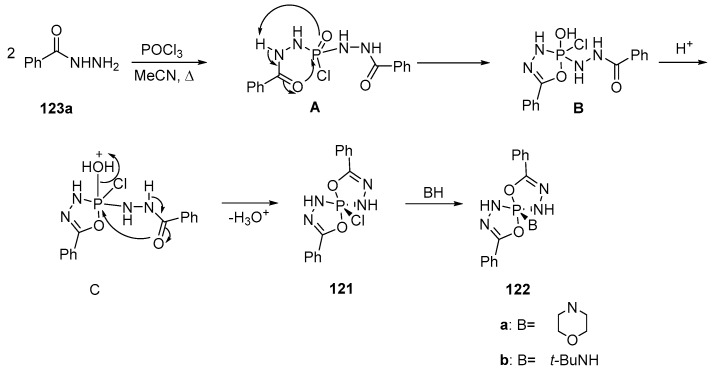
Syntheses of bicyclic diaminophosphoranes **121** and **122**.

The alternative approach to synthesize bicyclic pentacoordinate spirophosphoranes proposed by Gholivand et al. [106] is based on the condensation of simple hydrazides (i.e., benzhydrazide **123a** and 4-pyridinecarboxylic acid hydrazide **123b**) with phosphoryl reagents followed by a dehydration–cyclization rearrangement of the resulting phosphorylated hydrazides. Such spiro-bicyclophosphorane with a trigonal bipyramidal structure is formed if phosphoryl reagent contains at least two appropriate leaving groups such as chlorine atoms (in PhPOCl_2_ or POCl_3_). Benzhydrazide reacted with POCl_3_ in refluxing acetonitrile to give the P–Cl phosphorane intermediate **121** which treated with the appropriate amine was converted to the P-N spirophosphorane **122a**,**b** (Scheme 51). The mechanism proposed for the formation of the intermediate involved β-amidic proton elimination in phosphorylated hydrazides **A** upon which the cyclization product with new C=N imine bond **B** was formed. The intermediate was finally converted into **121** by dehydration.

**Scheme 52:**
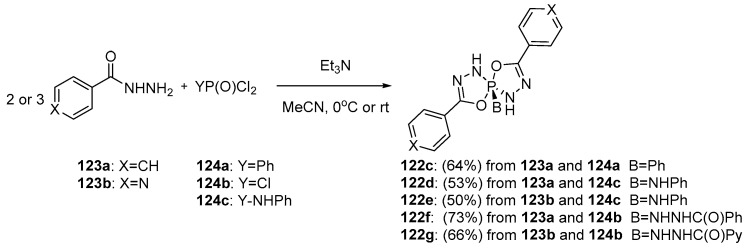
Synthesis of bicyclic diaminophosphoranes **122**.

Similarly, several pentacoordinate phosphoranes **122c**–**g** were easily prepared in the reaction of benzhydrazide **123a** or 4-pyridinecarboxylic acid hydrazide **123b** with PhPOCl_2_, PhNHPOCl_2_ or POCl_3_ in the presence of triethylamine [106] (Scheme 52). 

##### 10P-1C-4O Phosphoranes

**Scheme 53:**
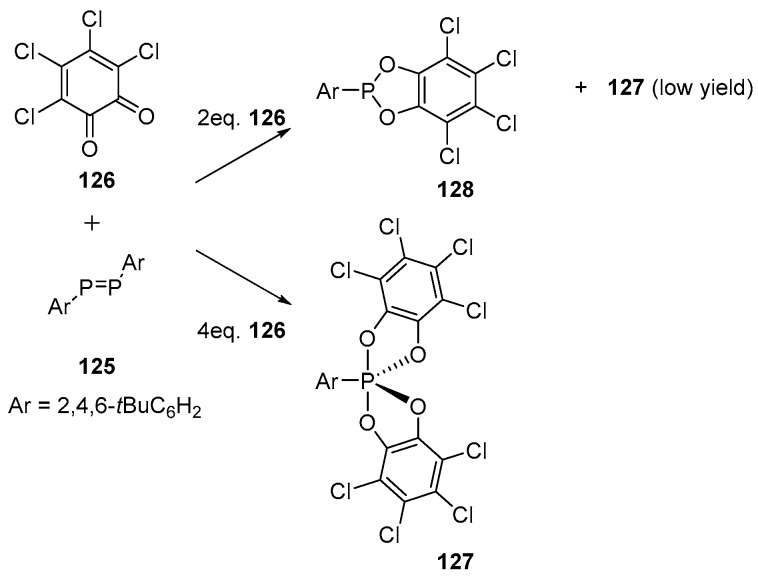
Synthesis of bicyclic phosphoranes **127**.

The reaction of (*E*)-bis-(2,4,6-tri-*t*-butylphenyl)diphosphane **125** with a fourfold excess of tetrachloro-*o*-benzoquinone **126** was found to give the chiral perchlorinated spirophosphorane **127**. It was suggested that the reaction, proceeding according to an electron transfer mechanism, occurred via **128** with a cleavage of the P-P bond and indeed, **127** was isolated in low yield even with two equivalents of **126** (Scheme 53) [107].

**Scheme 54:**
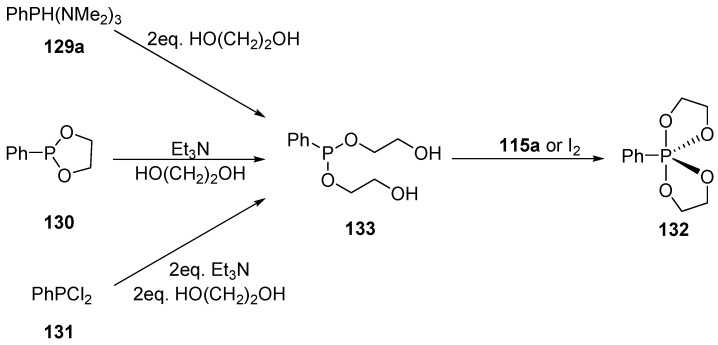
Synthesis of bicyclic phosphorane **132**.

Similarly, the reactions of tricoordinated organophosphorus precursors **129**–**131** with an appropriate equivalent of ethylene glycol was found to give the spirophosphorane **132**. All the three reactions occurred via the bis-ester **133** formed in a nucleophilic exchange reaction at the tricoordinated phosphorus center of precursors **129**–**131** [108] (Scheme 54).

##### 10P-1C-1N-2O-1H Phosphoranes

**Scheme 55:**
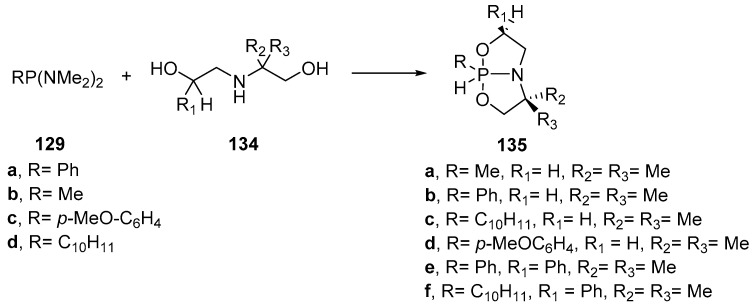
Synthesis of bicyclic azaphosphoranes **135**.

The reactions of tricoordinated phosphonous diamides **115a**–**d** with an appropriate equivalent of aminodiols **120** led to a series of chiral enantiomeric or diastereomeric bicyclic spirophosphoranes **121a**–**f** (Scheme 55) [109].

##### 10P-2N-2O-1H and 10P-2N-3O Phosphoranes

**Scheme 56:**
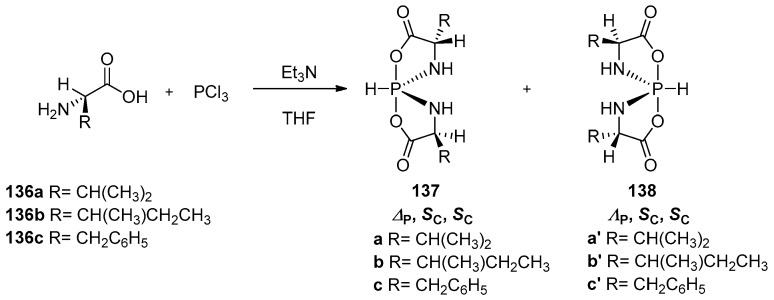
Synthesis of hydrospirophosphoranes **137** and **138** derived from l-amino acids.

Chiral hydrospirophosphoranes derived from *L*-amino acids were for the first time obtained by Koenig et al. as early as 1979 [110]. More recently this approach based on the reaction of phosphorus trichloride with amino acids was used by Zhao and coworkers for the preparation of spirophosphoranes from enantiomerically pure valine **136a**, isoleucine **136b** and phenylalanine **136c**. [111,112,113,114]. The spirophosphoranes formed as mixtures of diastereoisomers were separated to the pure stereoisomers **137a**–**c** and **138a**–**c** by reverse-phase HPLC or recrystallization (Scheme 56).

**Scheme 57:**
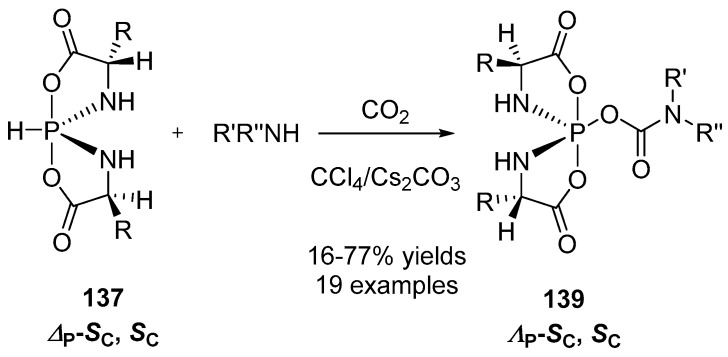
Preparation of pentacoordinate spirophosphorane carbamates **139**.

**Scheme 58:**
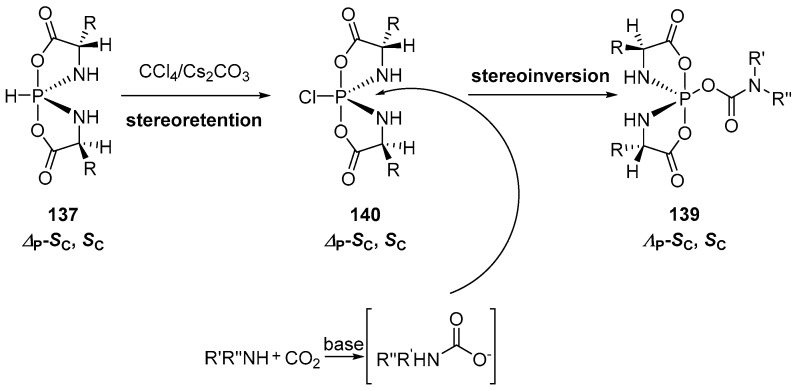
The mechanism of formation spirophosphorane carbamates **139**.

The pure stereoisomers **137** were used for the preparation of pentacoordinate spirophosphorane carbamates **139** [115] via the two-steps Atherton–Todd-type reaction (Scheme 57). Initially, the spirophosphoranes **137** reacted with CCl_4_ yielding chlorospirophosphoranes **140** (with retention of configuration at the phosphorus atom). In a second step chlorophosphoranes, upon the reaction with carbamate anions, (formed in situ from CO_2_ and secondary amines), afforded the carbamates **139** with inversion of configuration (Scheme 58).

**Scheme 59:**
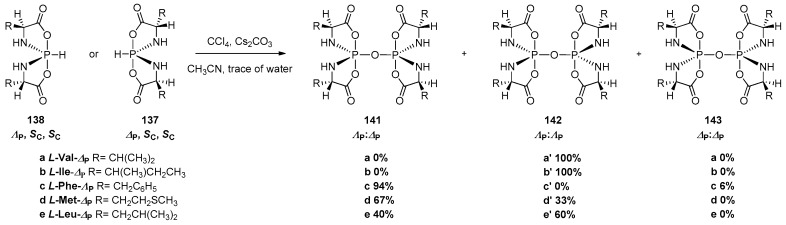
Synthesis of pentacoordinate pyrospirophosphoranes **141**–**143**.

Similarly, the spirophosphoranes **137a**–**e** and **138a**–**e** were used as substrates for the synthesis of pentacoordinate pyrospirophosphoranes containing a P–O–P bond **141**–**143** under modified Atherton–Todd conditions [116] (Scheme 59). It was found, upon optimization of the reaction conditions, that the spirophosphoranes **137a**,**b** gave the pure diasteroisomer of pyrospirophosphoranes **142a**,**b** whereas a mixture of diastereoisomers **141c**–**e** and **143c**–**e** were isolated if hydrospirophosphoranes **137c**–**e** were used as substrates.

**Scheme 60:**
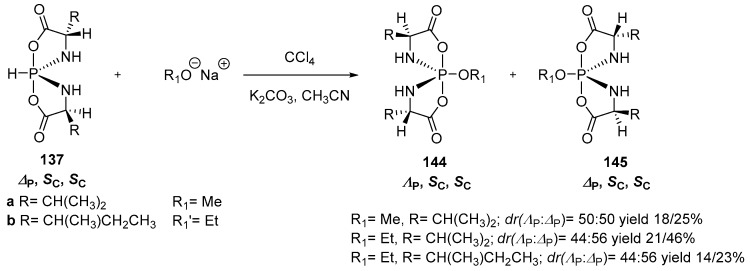
Synthesis of alkoxy spirophosphoranes **144**,**145**.

A series of the alkoxy spirophosphoranes **144**,**145** was also prepared by this approach (Scheme 60) but unfortunately as a mixture of diastereoisomers (in most cases in 1:1 ratio) [117].

**Scheme 61:**
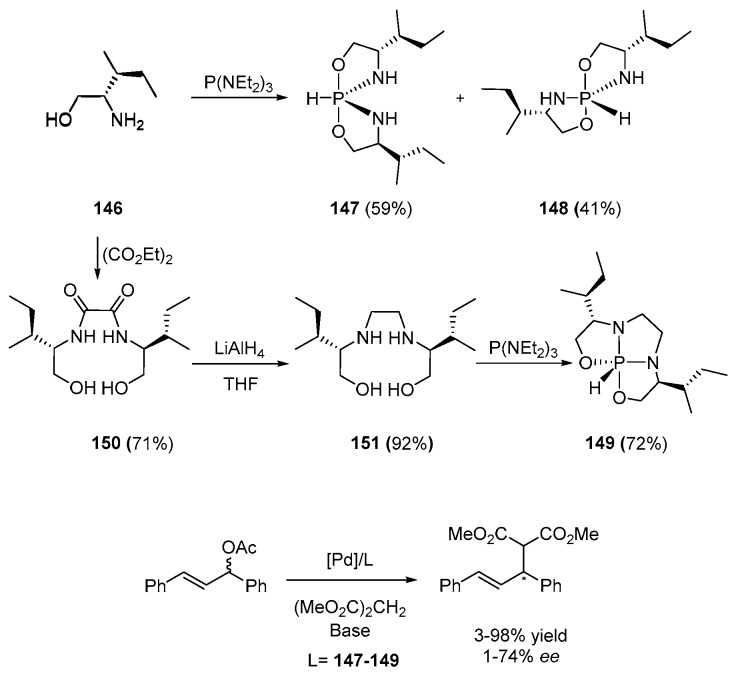
Syntheses of bicyclic hydrophosphoranes **147**–**149** and their use as chiral ligands.

Bicyclic hydrophosphoranes **147** and **148** were synthesized as a 3:2 mixture of two epimers by the reaction of tris-(*N*,*N*-diethyl)phosphorus amide with isoleucinol **146**. On the other hand, the tricyclic phosphorane **149** was isolated as a single stereoisomer in a similar reaction with the diaminodiole **151** (Scheme 61) [118]. The isolated spirophosphoranes **147**–**149** were used as chiral ligands in the Pd-catalyzed alkylation of 1,3-diphenyl allyl acetate with acceptable stereoselectivity (up to 74% *ee*) (Scheme 61) [119]. Moreover, these hydrophosphorane derivatives were used to obtain complexes with [Pt(COD)Cl_2_], [Rh(CO)_2_Cl]_2_ [Rh(THF)_2_(COD)]^+^BF_4_^−^.

**Scheme 62:**
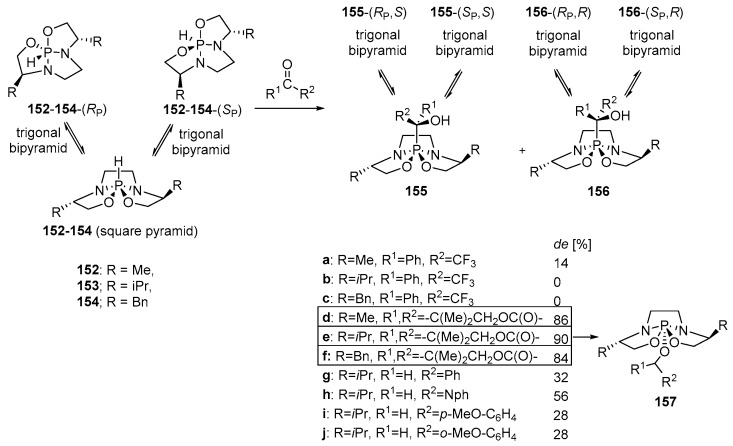
Chiral triquinphosphoranes **152**–**154** and their reactions with various activated carbonyl compounds.

Chiral triquinphosphoranes **152**–**154** were easily synthesized from chiral enantiopure diaminodiols with a C_2_ symmetry axis. It was shown that their structure is best represented by two trigonal bipyramid structures (TBP) with opposite absolute configurations at the phosphorus atom, *R*_P_ and *S*_P_, being in a fast equilibrium by a Berry pseudorotation process (Scheme 62) [120]. They reacted with various activated carbonyl compounds: trifluoroacetophenone, ketopantolactone and aromatic aldehydes to afford two diastereomeric hydroxyphosphoranes with a *de* up to 90% depending on the nature of the electrophile (Scheme 62). Diastereomeric mixture of hydroxyphosphoranes formed by the addition of ketopantolactone **155**–**156d**–**f** were quantitatively converted into alkoxyphosphoranes **157** with diastereomeric excesses decreasing from 86% to 8% for R = Me, from 90% to 50% for R = iPr and from 84% to 2% for R = Bn.

**Scheme 63:**
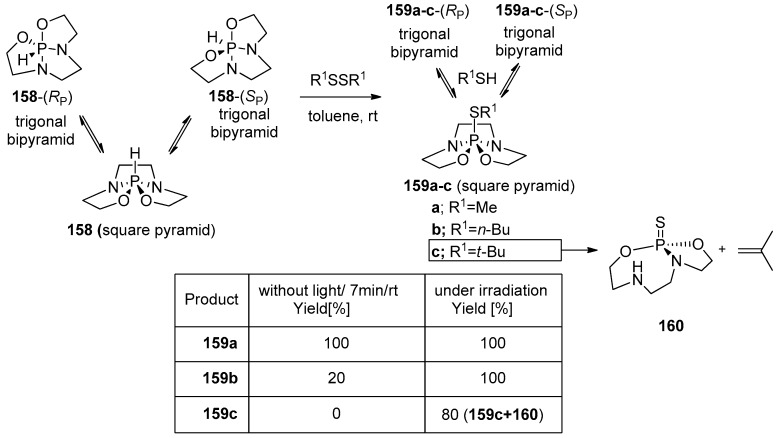
Reaction of chiral hydrido-triquinphosphorane **158** with alkyl disulfides.

Similarly, the parent triquinphosphorane **158** was found to react with methyl, *n*-butyl, or *t*-butyl disulfide to produce the corresponding alkylthiophosphoranes **159a**–**c**, although *t*-butylthio-phosphorane **159c** was only a minor product and the major one was identified as the thiophosphoramide **160** (Scheme 63). Under irradiation at −50 °C *t*-butylthiophosphorane **159c** was produced in high yield, however, by increasing the reaction temperature its slow conversion to **160** was observed. The reactivity of the triquinphosphorane **158** with alkyl disulfides was compared both under irradiation and in the dark at room temperature. The reactivity increased significantly in the case of *n*-butyl disulfide and *t*-butyl disulfide when the reactions were conducted under irradiation. This observation led to the conclusion that this type of the reaction does involve free radical species [121].

##### 10P-1N-3O-1H and 10P-2N-2O-1H Phosphoranes

**Scheme 64:**
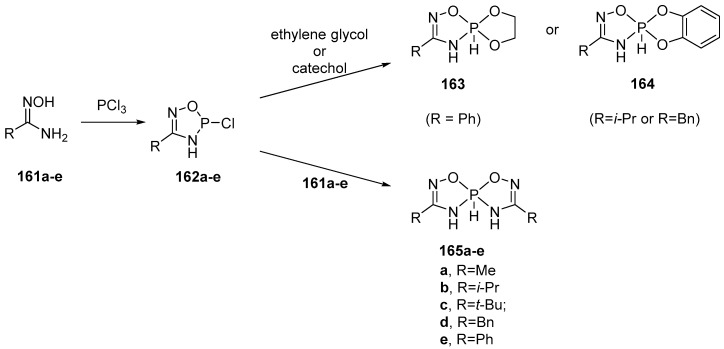
Syntheses of bicyclic hydrophosphoranes **163**–**165**.

Three synthetic protocols were applied to the preparation of this type of hydrophosphoranes with the use of amidooxime derivatives as substrates [122]. The first procedure started with the reaction of amidooxime **161a**–**e** with PCl_3_ leading to 5-chloro-1,2,4,5-oxadiazaphospholines **162a**–**e**. Their reactions with ethylene glycol or *ortho*-hydroxyphenol afforded the unsymmetrical spirophosphoranes **163** or **164** whereas the treatment with another dose of the amidooxime **161** led to symmetrically substituted spirophosphoranes **165a**–**e** (Scheme 64).

**Scheme 65:**
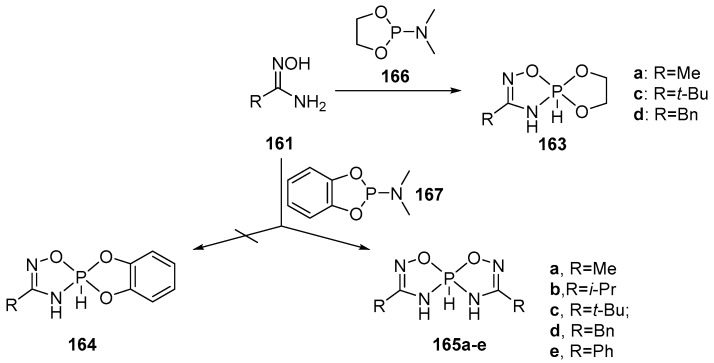
Syntheses of bicyclic hydrophosphoranes **163** and **165**.

In the second approach, amidooxime **161a**,**c**,**d** upon reaction with 2-dimethylamino-1,3,2-dioxaphospholane **166** provided unsymmetrical spirophosphoranes **163a**,**c**,**d** and in the reaction with 2-dimethylamino-(4,5)benzo-1,3,2-dioxaphospholane **167** in acetonitrile yielded unexpected symmetrically substituted spirophosphoranes **165a**–**e** instead of unsymmetrically substituted phosphorane **164a**–**e** (Scheme 65).

**Scheme 66:**
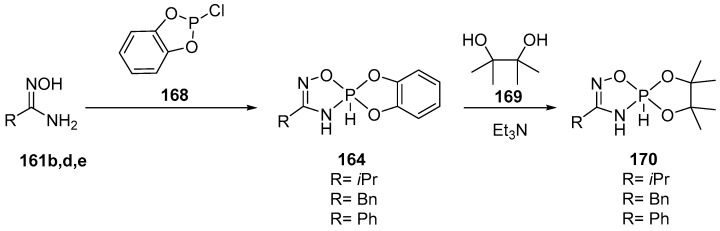
Synthesis of bicyclic hydrophosphoranes **164** and **170**.

The third synthetic pathway involved the treatment of amidooximes **161b**,**d**,**e** with 2-chloro-(4,5)benzo-1,3,2-dioxaphospholane **168** leading, as it was expected, to the corresponding unsymmetrical spirophosphoranes **164**. The latter were converted into the corresponding unsymmetrical spirophosphorane **170** by transesterification with pinacol **169** taking place in the presence of triethylamine (Scheme 66).

##### 10P-2N-3O and 10P-1N-4O Phosphoranes

**Scheme 67:**
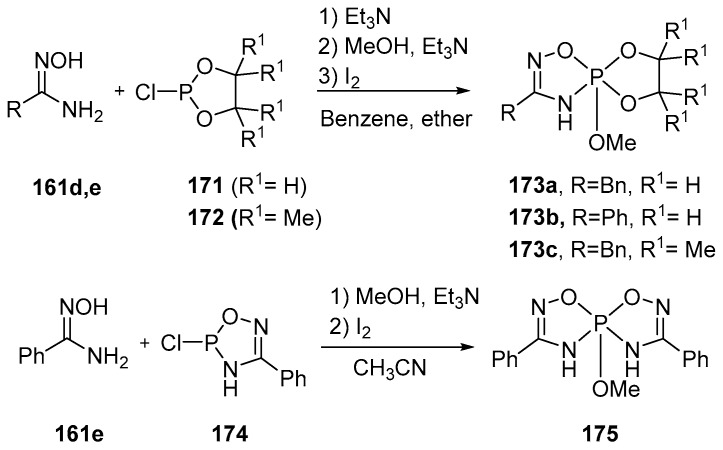
Synthesis of bicyclic spirophosphoranes **173** and **175**.

Amidooximes **161d**,**e** were used also as substrates for the preparation of the spirophosphoranes containing the P–OMe grouping **173**, **175**. They were isolated upon application of the oxidation procedure carried out under basic conditions with iodine as an oxidant [122] (Scheme 67).

**Scheme 68:**
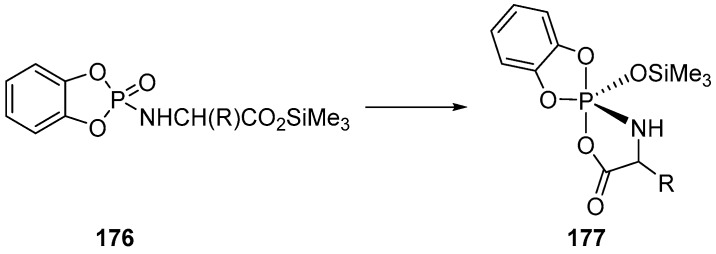
Synthesis of bicyclic phosphoranes **177**.

*O*-Trimethylsilyl derivatives of spirophosphoranes **177** containing an amino acid residue were formed by the cyclisation of the corresponding cyclic amides **176** [123] (Scheme 68). The resulting spirophosphoranes **177** were used for the formation of peptides from amino acids such as histidine, serine, threonine and α-alanine but not β-alanine.

**Scheme 69:**
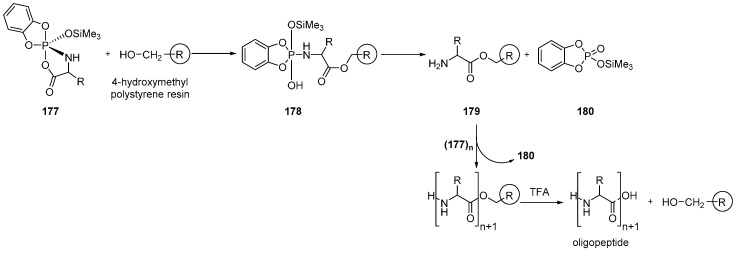
Synthesis of oligopeptides with the use of spirophosphoranes **177** as substrates.

Later on, spirophosphoranes **177** were also used as substrates in a new method for the solid phase synthesis of oligopeptides. This approach was based on their rapid reaction with hydroxymethyl polystyrene resin leading to unstable hydroxyphosphoranes **178** which after mild hydrolysis gave the phosphate **180** and a solid phase bound amino-acids **179**. Coupling of **179** with further phosphorane **177**, followed by hydrolysis afforded a solid phase bound dipeptide. The process was repeated to give oligopeptides. The final oligopeptide was liberated from the resin by treatment with TFA [124] (Scheme 69).

##### 10P-4O-1H Phosphoranes

**Scheme 70:**
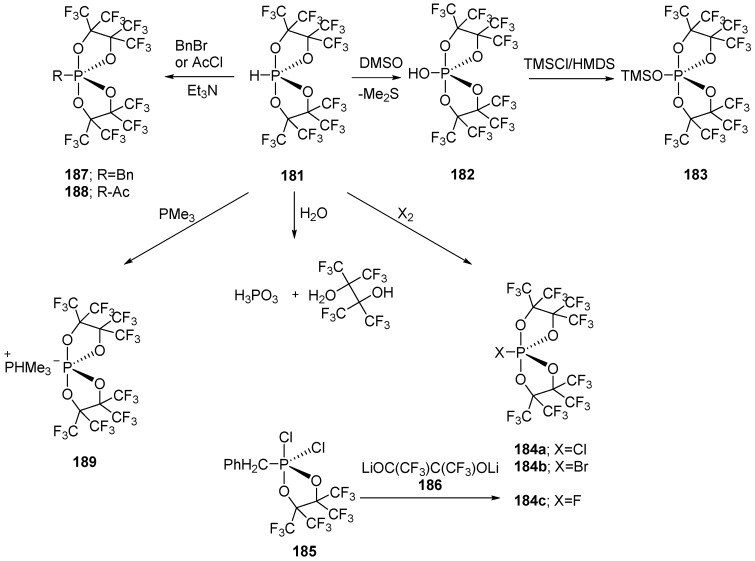
Interconversions of spirophosphoranes **181** and **182**.

The synthesis and reactivity of the hydrophosphorane **181** derived from perfluoropinacol was described as early as 1983 [125]. It was found that it could be rapidly oxidized by dimethyl sulfoxide to form the hydroxyphosphorane **182**, and then silylated to the derivative **183**. Chlorine and bromine reacted with **181** to give the corresponding halospirophosphoranes **184a**,**b**. In this context it is interesting to note that the corresponding fluorospirophosphorane **184c** was obtained from the dichlorofluoro-perfluoropinacolophosphorane FCl_2_P(CH_2_Ph)[OC(CF_3_)C(CF_3_)O] **185** and dilithium perfluoropinacolate LiOC(CF_3_)C(CF_3_)OLi **186**. In the presence of triethylamine the hydrophosphorane **181** reacted with benzyl bromide or acetyl chloride to form the phosphoranes **187** or **188**. Moreover, the treatment of the hydrophosphorane **181** with trimethylphosphine gave a thermally unstable phosphonium salts **189**. Hydrophosphorane **181** undergoes fast hydrolysis affording phosphorous acid and perfluoropinacol [125] (Scheme 70).

##### 10P-4O-1Metal and 10P-3N-1O-1Metal Phosphoranes

**Scheme 71:**
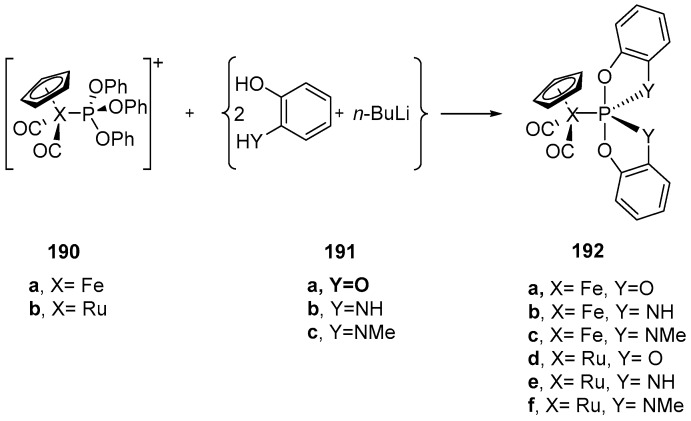
Preparation of chiral metallophosphoranes **192**.

Another group of chiral pentacoordinate organophosphorus compounds are metallophosphoranes **192a**–**e**. Nakazawa et al. [126] developed a synthetic method for the preparation of metallophosphoranes **192**, which was based on the nucleophilic attack of the, in situ generated, anion **191a**–**e** on the complexed trivalent phosphorus atom **190a**,**b** (Scheme 71). For example, ferrocenyl complex **190a** reacted with in situ generated anions **191a**–**e** to form chiral metallophosphoranes **192a**–**e**. The valency of the phosphorus atom increased from III to V without breaking the Fe-P bond and the nature of Fe-P bond changed from coordinative to covalent.

**Scheme 72:**
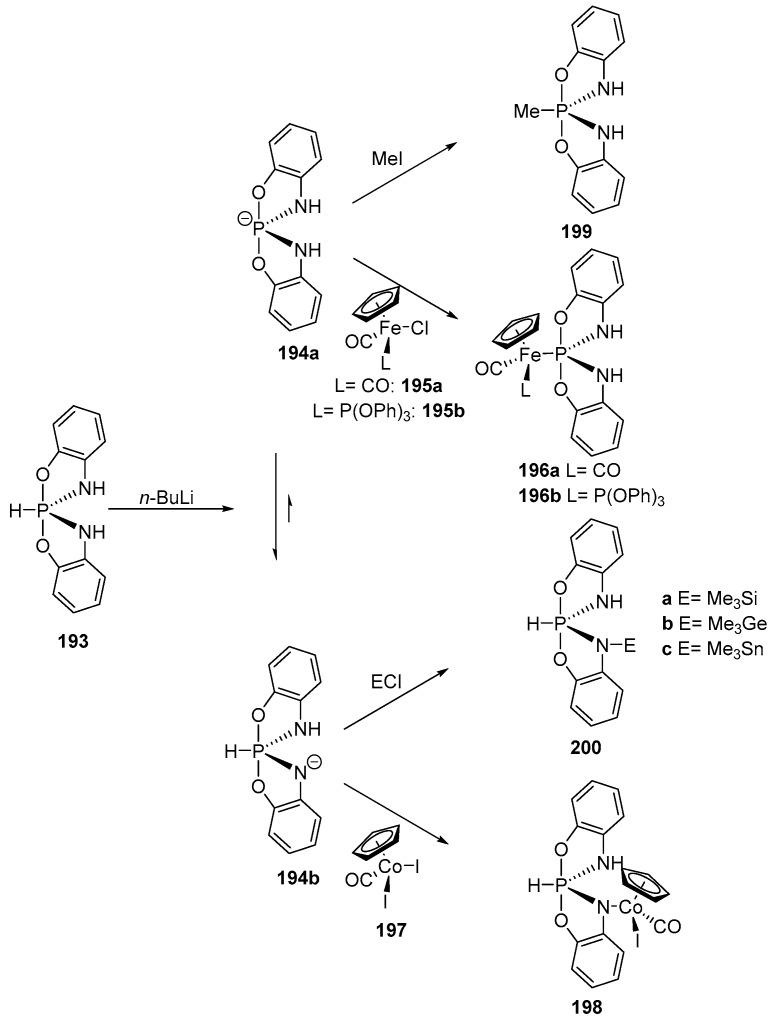
Functionalization of hydrophosphorane **193** leading to phosphoranes **196** and **198**–**200**.

It is worthy to note that treatment of HP(C_6_H_4_NH)_2_-**193** with *n*BuLi led to unexpected deprotonation on nitrogen atom (giving **194b**) instead on phosphorus (to afford **194a**). Moreover, an amide anion **194b** is in an equilibrium with phosphoranide **194a**. The reaction of the in situ generated anion **194a** with [Cp(CO)LFeCl] (**195a** or **195b**) gave a P-metallated phosphorane **196a** or **196b**. On the contrary, the reaction the equilibrated mixture of **194** with [Cp(CO)CoI_2_]-**197** led to *N*-metallated compound **198**. Interestingly, in the reaction of the phosphoranide **194a** with MeI the P-methylated product **199** was formed. On the other hand, electrophiles such as Me_3_SiCl, Me_3_GeCl or Me_3_SnCl, provided *N*-substituted products (**200a**–**c**) [127,128] (Scheme 72).

**Scheme 73:**
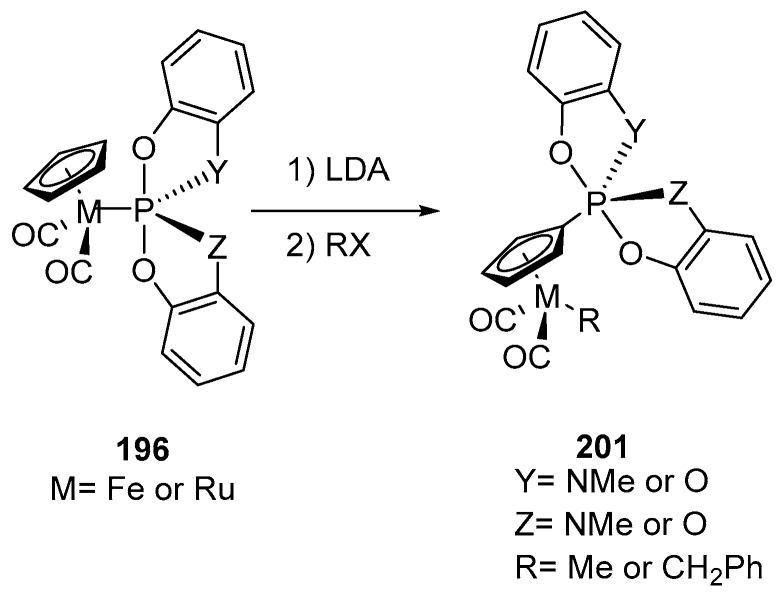
Alkylation of metallophosphoranes **196**.

Moreover, it was found that in the presence of a base a migration of pentacoordinate phosphorane fragment occured from the transition metal in **196** to the carbon atom in the Cp ring in **201** [129] (Scheme 73).

##### 10P-5O Phosphoranes

**Scheme 74:**
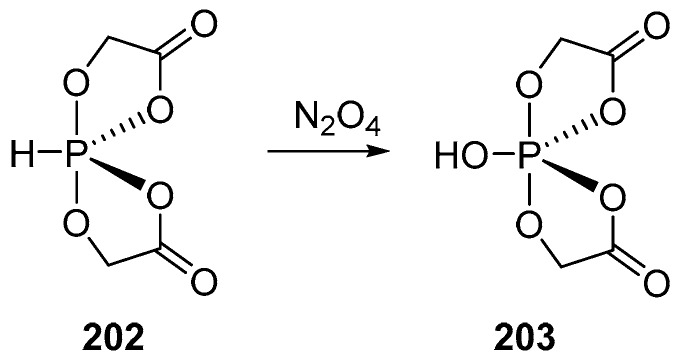
Oxidation of hydrophosphorane **202**.

The hydroxyphosphorane **203** was prepared by N_2_O_4_ oxidation of the parent hydrophosphorane **202** (Scheme 74). Its single crystal analysis showed an almost perfect trigonal bipyramidal structure with the unit cell containing two molecules of the same helicity connected by H-bonds between the P–OH and carbonyl groups [130].

**Scheme 75:**
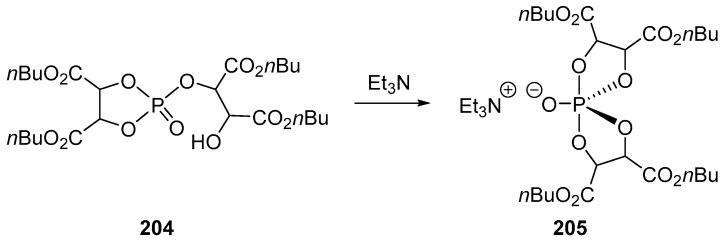
Formation of salt **205**.

The triethylammonium salt of the hydroxyspirophosphorane **205** derived from *n-*butyl tartrate was prepared on treatment of the very acidic [p*K*_a_ = 4.4(DMSO)] monocyclic phosphorus ester **204** with triethylamine (Scheme 75) [131].

**Scheme 76:**
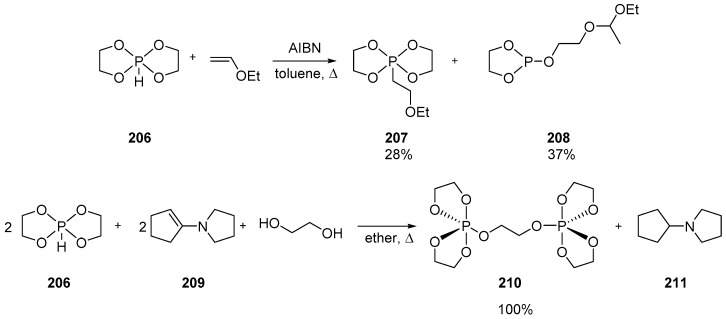
Synthesis of spirophosphoranes **207** and **210.**

The radical reaction of the bicyclic hydrophosphorane **206** derived from glycol with ethyl vinyl ether provided the corresponding P–C spirophosphorane **207** along with the monocyclic phosphite **208**. The reaction of the hydrospirophosphorane **206** with ethylene glycol and enamine **209** afforded the pentaoxyspirophosphorane **210** with the simultaneous reduction of the enamine **209** to amine **211** [132] (Scheme 76).

## 3. Conclusions

Enantiomerically enriched pentavalent phosphoranes constitute an interesting group of heterocyclic chiral auxiliaries. Their still limited applications in asymmetric synthesis are however hampered mainly by a limited access to diastereomerically pure and especially enantiomerically pure (or at least enantiomerically enriched) samples. It is our hope that this review, which describes briefly the basic procedures used for the preparation of these derivatives, their stereoisomerization mechanisms and their selected interconversions, will encourage wider interest in the synthesis, structural determinations and applications of these groups of chiral, hypervalent organophosphorus derivatives.

## Figures and Tables

**Table 1:** Number of chiral and achiral species of 10-P-5 (TBP geometry).

General Structure	Number of Different Structures and Their Chirality
PL_5_	1 achiral
PL^1^_4_L^2^	2 achiral
PL^1^_3_L^2^_2_	3 achiral
PL^1^_3_L^2^L^3^	2 achiral and 2 pairs of enantiomers
PL^1^_2_L^2^_2_L^3^	2 achiral and 3 pairs of enantiomers
PL^1^_2_L^2^L^3^L^4^	1 achiral and 6 pairs of enantiomers
PL^1^L^2^L^3^L^4^L^5^	10 pairs of enantiomers
